# Stimulating Neoblast-Like Cell Proliferation in Juvenile *Fasciola hepatica* Supports Growth and Progression towards the Adult Phenotype *In Vitro*

**DOI:** 10.1371/journal.pntd.0004994

**Published:** 2016-09-13

**Authors:** Paul McCusker, Paul McVeigh, Vignesh Rathinasamy, Hayley Toet, Erin McCammick, Anna O’Connor, Nikki J. Marks, Angela Mousley, Gerard P. Brennan, David W. Halton, Terry W. Spithill, Aaron G. Maule

**Affiliations:** 1 Microbes & Pathogen Biology, The Institute for Global Food Security, School of Biological Sciences, Queen’s University Belfast, Belfast, United Kingdom; 2 Department of Animal, Plant and Soil Sciences, and Centre for AgriBioscience, La Trobe University, Bundoora, Australia; University of Melbourne, AUSTRALIA

## Abstract

Fascioliasis (or fasciolosis) is a socioeconomically important parasitic disease caused by liver flukes of the genus *Fasciola*. Flukicide resistance has exposed the need for new drugs and/or a vaccine for liver fluke control. A rapidly improving ‘molecular toolbox’ for liver fluke encompasses quality genomic/transcriptomic datasets and an RNA interference platform that facilitates functional genomics approaches to drug/vaccine target validation. The exploitation of these resources is undermined by the absence of effective culture/maintenance systems that would support *in vitro* studies on juvenile fluke development/biology. Here we report markedly improved *in vitro* maintenance methods for *Fasciola hepatica* that achieved 65% survival of juvenile fluke after 6 months in standard cell culture medium supplemented with 50% chicken serum. We discovered that this long-term maintenance was dependent upon fluke growth, which was supported by increased proliferation of cells resembling the “neoblast” stem cells described in other flatworms. Growth led to dramatic morphological changes in juveniles, including the development of the digestive tract, reproductive organs and the tegument, towards more adult-like forms. The inhibition of DNA synthesis prevented neoblast-like cell proliferation and inhibited growth/development. Supporting our assertion that we have triggered the development of juveniles towards adult-like fluke, mass spectrometric analyses showed that growing fluke have an excretory/secretory protein profile that is distinct from that of newly-excysted juveniles and more closely resembles that of *ex vivo* immature and adult fluke. Further, *in vitro* maintained fluke displayed a transition in their movement from the probing behaviour associated with migrating stage worms to a slower wave-like motility seen in adults. Our ability to stimulate neoblast-like cell proliferation and growth in *F*. *hepatica* underpins the first simple platform for their long-term *in vitro* study, complementing the recent expansion in liver fluke resources and facilitating *in vitro* target validation studies of the developmental biology of liver fluke.

## Author Summary

Parasitic worms require a host organism in order to survive and reproduce. As such, it is difficult to study them outside of a host. Some parasites can be maintained *in vitro* using cell culture methods; in the case of *F*. *hepatica*, previously-reported methods are unsatisfactory because they are difficult to reproduce and unable to support long term growth and development. Here we have developed a new set of methods for maintaining *F*. *hepatica* juveniles *in vitro*. These methods use simple, commonly available reagents and techniques, enabling us to keep fluke alive *in vitro* for at least 6 months, as well as stimulating the development of characteristics that resemble adult parasites. Over time, our *in vitro* fluke show changes in the structure and complexity of individual tissues, and the proteins they produce, such that they are more reminiscent of adult, than juvenile fluke. Additionally, we demonstrate that fluke growth is supported by the division of cells resembling stem cells, which have not been reported previously for *F*. *hepatica*. This work will support the study of liver fluke, enabling the development of new drugs and vaccines for the treatment of liver fluke infections of humans and animals.

## Introduction

Fascioliasis (or fasciolosis), a parasitic disease caused by liver flukes of the genus *Fasciola*, has significant economic and animal health impacts on the global agri-food industry. Global economic losses due to fascioliasis are estimated at around US$3.2 billion annually [1], although a more recent study has identified impacts of up to US$4.78 billion in India alone [2], while in the UK, fascioliasis costs the agri-food sector around £300 million [3]. *Fasciola hepatica* infection has become increasingly prevalent in humans, with up to 17 million people infected and 91 million at risk worldwide [4], such that human fascioliasis is considered a Neglected Tropical Disease of major global and regional importance by the World Health Organization. Infection levels are set to rise further with increasing levels of resistance to triclabendazole (the current drug of choice) [5,6] and a potential explosion of fluke populations due to climate change [7]. Therefore, there is a pressing need to identify and evaluate novel diagnostic, therapeutic and preventative options for fascioliasis.

Helminth parasitology has benefited from recent advances in transcriptomic, genomic and functional-genomic resources. These additions to the helminth ‘molecular toolbox’ have enhanced our ability to probe the fundamental biology of, and identify and validate therapeutic targets in, parasitic helminths. The *F*. *hepatica* toolset currently consists of a draft genome [8], several developmentally-staged transcriptomes [9,10] and the (RNA interference (RNAi) tools with which to functionally interrogate these datasets [11–14]. Similarly, advanced proteomics and sub-proteomic methods provide tools for advancing our understanding of fluke virulence and the host-parasite interface [15]. However, the effective use of these tools has been hindered by the absence of an effective *in vitro* maintenance system for *F*. *hepatica* juveniles and immature life stages (the most pathogenic life stage of the fluke). In contrast, schistosome blood flukes can be maintained quite simply *in vitro* for many months in serum-supplemented culture medium, a method that has supported several functional genomic studies in *Schistosoma mansoni* [16] and *S*. *japonicum* [17], while similarly simple methods have supported RNAi studies in *Opisthorchis viverrini* [18] and *Clonorchis sinensis* [19].

All of the reported *F*. *hepatica* culture methods are complex and of arguable utility, issues which have undoubtedly limited their adoption by the research community. Seminal studies from the 1960/70s reported successful *in vitro* maintenance of juvenile *F*. *hepatica* on a food source of cultured mammalian cell monolayers [20–22], but there were notable discrepancies between these studies. However, in the late 1970s a comprehensive study by Davies and Smyth [23] evaluated 39 different combinations of basic media variously supplemented with animal sera and blood. The most effective combination in this study was NCTC 135 medium supplemented with 20% chick serum and ~0.01% sheep red blood cells, a combination which supported fluke growth, yielding development of reproductive tissue after 12 days *in vitro*. However, the authors stated that this outcome was only seen on one occasion and could not be repeated with different batches of serum. A subsequent study found that 50% human serum in RPMI medium with 2% human red blood cells promoted *in vitro* survival of up to 14 weeks, over which time the development of reproductive systems was observed, although no images were shown, and only limited quantitative data were presented to support these observations [24]. Similar inconsistencies exist regarding the most effective serum for inducing growth, with Davies and Smyth [23] favouring chick serum over human serum whilst Smith and Clegg [24] took the opposite stance. The latter study measured only the six fastest growing juveniles in each media tested [24] and did not consider the variation in growth capacity between individuals.

These disparities and inconsistencies within and between studies have hindered the widespread uptake and refinement of *in vitro* maintenance methods for *F*. *hepatica*. As a result, experiments on *F*. *hepatica* juveniles and adults have tended to be performed over periods of hours or a few days. Consequently, studies of fluke gene expression and proteomics have been limited, with developmental studies only possible using non-contiguous systems, for example by comparing newly excysted juveniles (NEJs) grown *in vitro* with *ex vivo* liver-stage parasites and/or adults [25–27].

Here we have set out to develop a simple *in vitro* maintenance system for *F*. *hepatica* juveniles, using commonly available culture reagents. Our methods facilitate the survival of juvenile fluke *in vitro* for at least 6 months, during which time we observe rapid and consistent growth of newly-excysted juvenile fluke. These growing fluke exhibit the development of adult-like characteristics including reproductive structures, ultrastructural changes in the tegument towards an adult-like form, and an excretory/secretory (E/S) protein profile that is more reminiscent of immature liver or adult-stage fluke, than newly-excysted juveniles. We also demonstrate for the first time that *F*. *hepatica* growth and development is supported by the proliferation of neoblast-like stem cells. The methods that we describe permit long-term *in vitro* maintenance and development of *F*. *hepatica* juveniles to more mature forms and readily facilitate the study of developmental and temporal changes in biological parameters consistent with reduced animal use. These methods will support the exploitation of liver fluke poly-omics datasets in functional studies to interrogate gene function and seed the development of new drugs and vaccines. The work reported here also encourages the reduction of host animal use in studies of *F*. *hepatica* biology.

## Methods

For convenience we use the following definitions for fluke life stages: NEJ, newly excysted juvenile worms within 24 h of excystment and before they have been placed into maintenance media; juvenile, worms maintained *in vitro* in maintenance media; immature, migratory stage worms found in the liver parenchyma, *in vivo* (also referred to as 21 day worms); adult, reproductively mature worms found in the bile duct of the liver *in vivo*.

### Maintenance and growth measurement of *F*. *hepatica*

**Fig 1:**
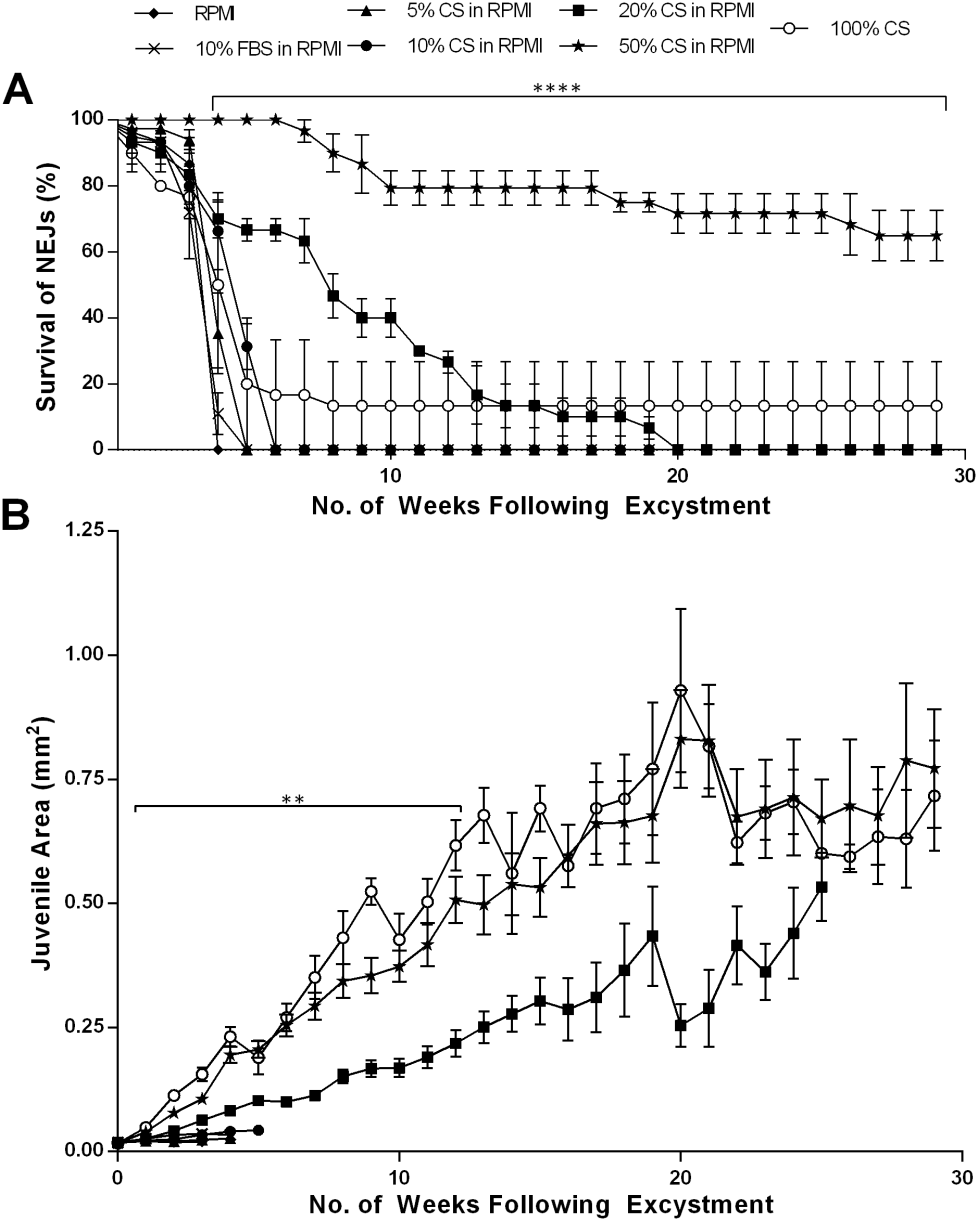
Survival and growth of juvenile *Fasciola hepatica* in vitro over 29 weeks following excystment. Juvenile fluke were maintained in: RPMI; 10% Foetal Bovine Serum (FBS) in RPMI; 5%, 10%, 20% or 50% Chicken Serum (CS) in RPMI; and, 100% CS. A—Percentage survival of juvenile *F*. *hepatica* over 29 weeks (mean±SEM). Statistical analyses were performed using One Way ANOVA with Dunnett’s post hoc test. ****, P<0.0001. B—Surface area of juvenile *F*. *hepatica* in mm^2^ (mean±SEM). Statistical analyses were performed using Kruskal-Wallis with Dunn’s post hoc test and One Way ANOVA with Dunnett’s post hoc test. **, P<0.01.

**Fig 2:**
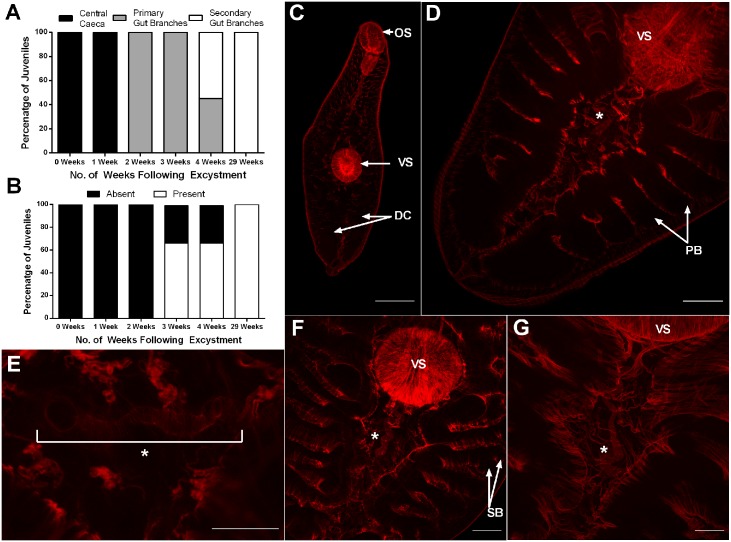
Development of digestive and reproductive tissues in *Fasciola hepatica* juveniles grown *in vitro*. Fluke were maintained in 50% Chicken Serum in RPMI. A—Percentage of juveniles exhibiting different degrees of gut branching at various times post-excystment; B—Percentage of juveniles with evidence of uterine tubing at various times post-excystment; C—Confocal microscope image of newly excysted juvenile exhibiting suckers (oral (OS) and ventral (VS)) and early digestive caeca (DC) where red is indicative of muscle actin staining) (scale bar 50 μm); D—Confocal microscope image of juvenile 3 weeks post-excystment exhibiting primary branching of the digestive caeca (PB) and early uterine tubing (*) (scale bar 50 μm); E—Confocal microscope image of uterine tubing (*) seen in a juvenile 3 weeks post-excystment (scale bar 25 μm); F—Confocal microscope image of juvenile 4 weeks post-excystment showing more pronounced uterine tubing (*) and secondary branching of the digestive caeca (SB) (scale bar 50 μm); G—Confocal microscope image of juvenile 29 weeks post-excystment revealing considerable growth of the juvenile and much extended uterine tubing (*) that proceeds underneath the ventral sucker (VS) (scale bar 50 μm).

**Fig 3:**
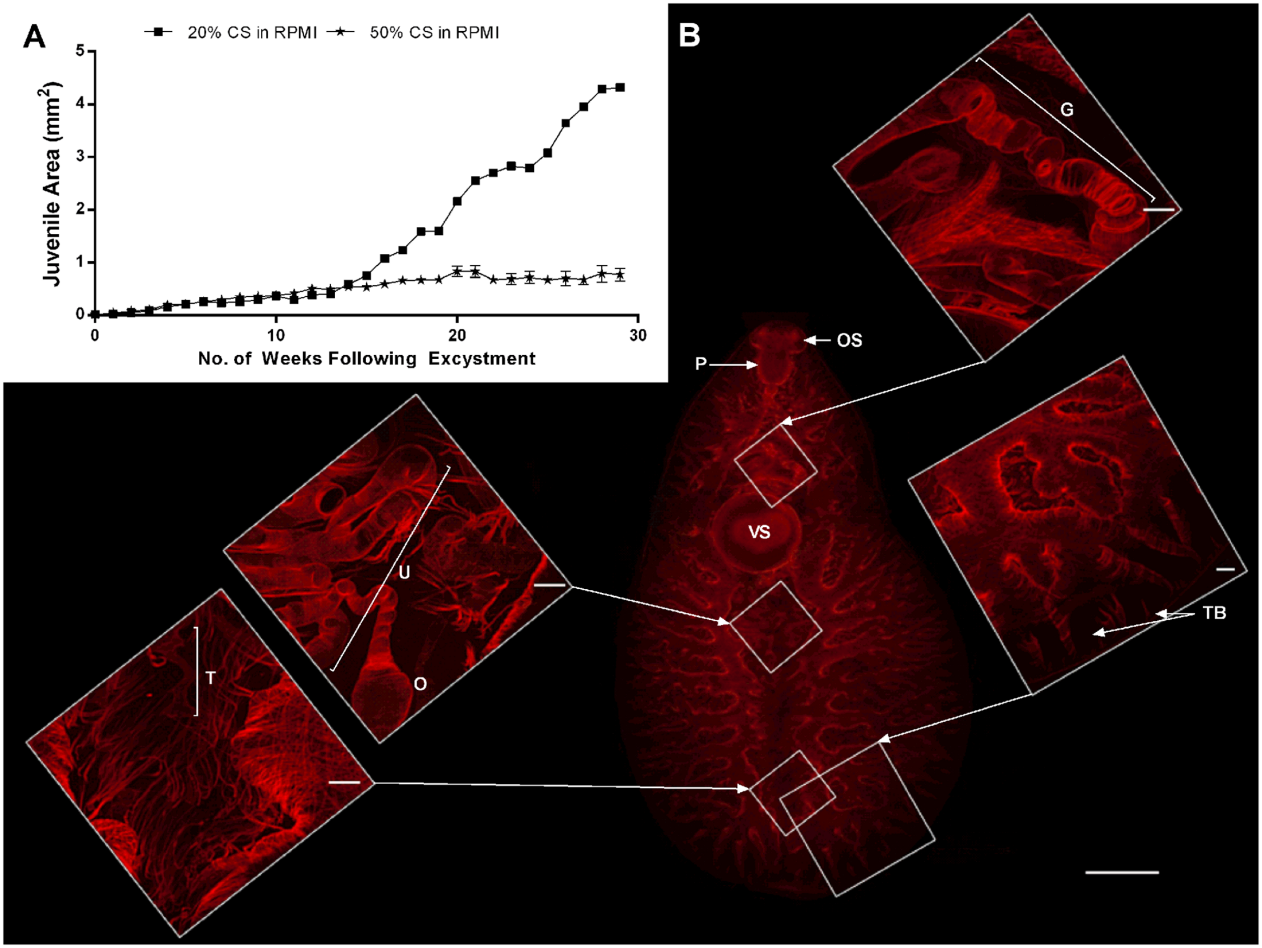
Growth and development of a rapidly growing juvenile *Fasciola hepatica* maintained *in vitro* (20% Chicken Serum [CS] in RPMI over 29 weeks). A—Growth of juvenile liver fluke maintained in 20% CS in RPMI or in 50% CS in RPMI; B—Confocal microscope images of the rapidly growing juvenile exhibiting oral sucker (OS), pharynx (P), gonopore tubing (G), ventral sucker (VS), uterine tubing (U), ootype (O), testes tubing (T) and tertiary branching of digestive caeca (TB) (scale bars 500 μm on main image, 50 μm on smaller, higher magnification images).

**Fig 4:**
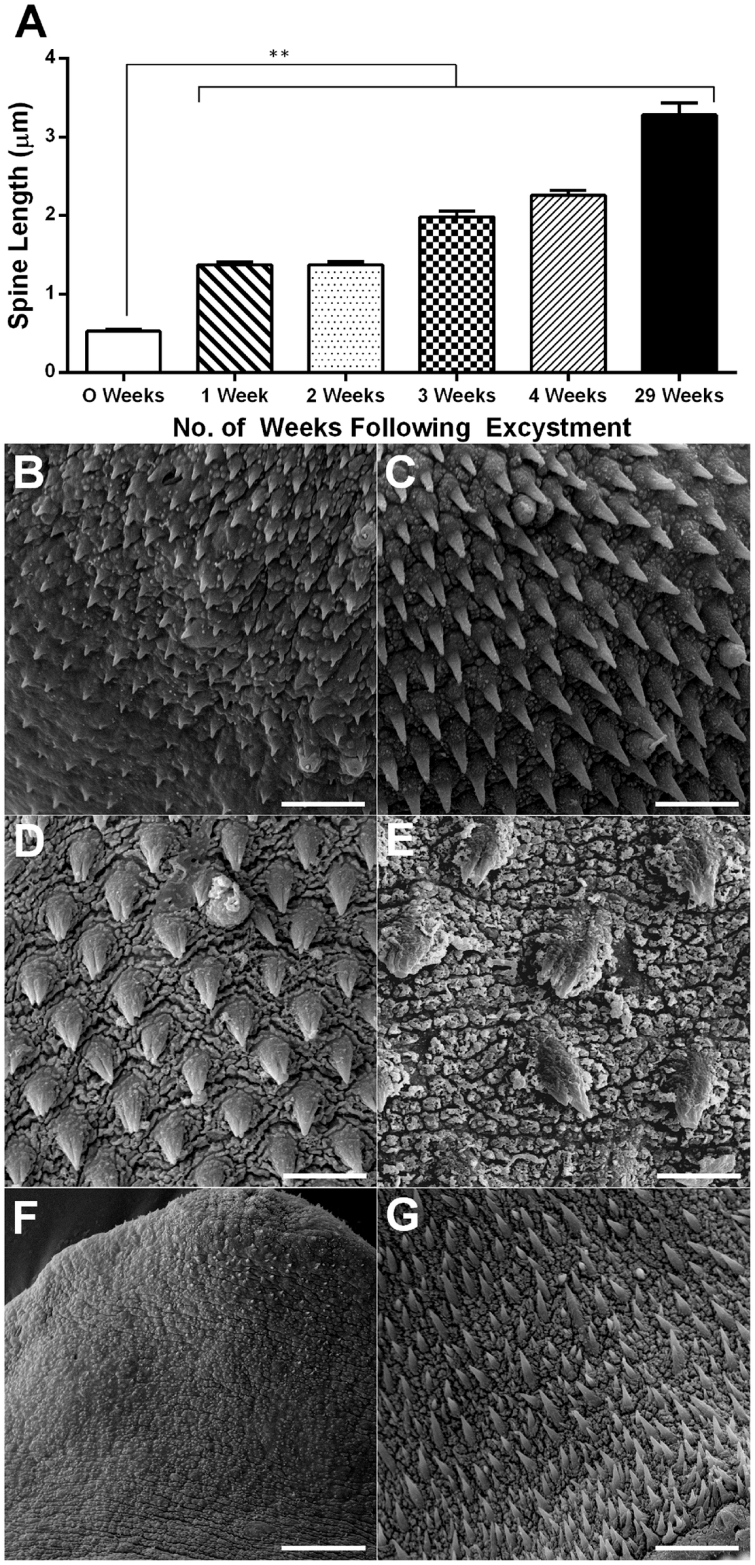
Development of tegument surface in *Fasciola hepatica* juveniles grown *in vitro* (in 50% Chicken Serum in RPMI). A—Change over time post-excystment in mean length of spines surrounding oral sucker of juveniles. Statistical analyses were performed using Kruskal-Wallis with Dunn’s post-hoc test, ** P<0.01; B—SEM image showing underdeveloped spines located between oral and ventral sucker in NEJ (scale bar 5 μm); C—Scanning Electron Microscope (SEM) image showing spines developing between oral and ventral sucker in juvenile 2 weeks post-excystment (scale bar 5 μm); D—SEM image showing spines developing two-tipped points between oral and ventral sucker in juvenile 4 weeks post-excystment (scale bar 5 μm); E—SEM image showing spines exhibiting multi-tipped points on anterior dorsal surface of juvenile 29 weeks post-excystment (scale bar 5 μm); F—SEM image showing absence of spines at posterior ventral surface of NEJ (scale bar 10 μM); G—SEM image showing appearance of developed spines at posterior ventral surface in juvenile 2 weeks post-excystment (scale bar 10 μm).

**Fig 5:**
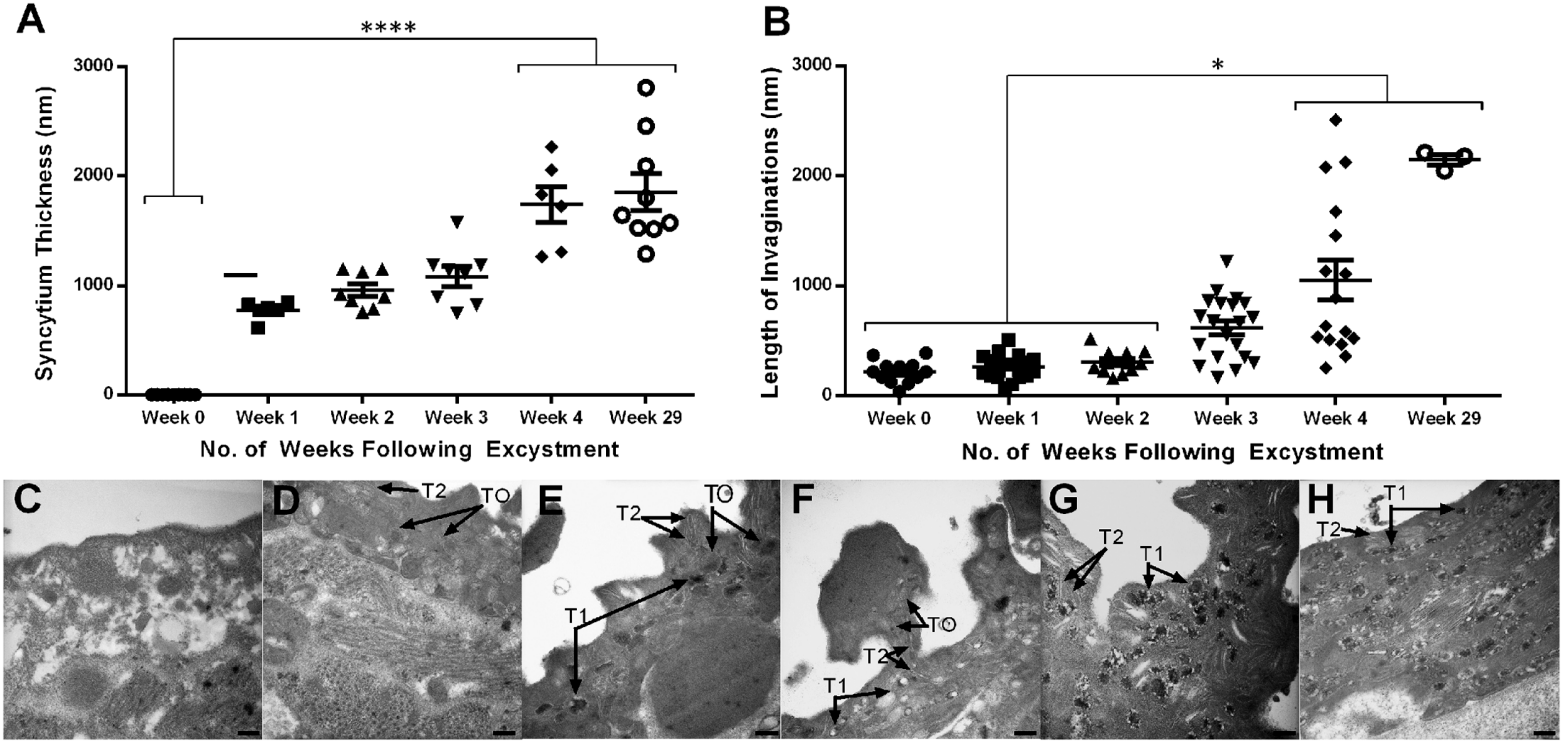
Ultrastructure of the developing tegument in juvenile *Fasciola hepatica* maintained *in vitro* (in 50% Chicken Serum in RPMI). A—Change over time post-excystment in mean tegument syncytium thickness of juveniles. Statistical analyses were performed using Kruskal-Wallis with Dunn’s post-hoc test, **** P<0.0001; B—Change over time post-excystment in mean tegument invagination length in juveniles. Statistical analyses were performed using Kruskal-Wallis with Dunn’s post-hoc test, * P<0.05; C—TEM image showing tegument of NEJ with few discernible features and no obvious syncytium; D—TEM image showing the tegument of a juvenile 1 week post-excystment with some T0 bodies and T2 bodies; E—TEM image showing the tegument of a juvenile 2 weeks post-excystment with the presence of both T0 bodies, early T1 bodies and T2 bodies; F—TEM image showing the tegument of a juvenile 3 weeks post-excystment with T0, T1 and T2 bodies present; G—TEM image showing the tegument of a juvenile 4 weeks post-excystment with mature T1 bodies visible and T2 bodies; H—TEM image showing tegument of juvenile 29 weeks post-excystment packed with mature T1 bodies and T2 bodies. All scale bars, 500 nm.

**Fig 6:**
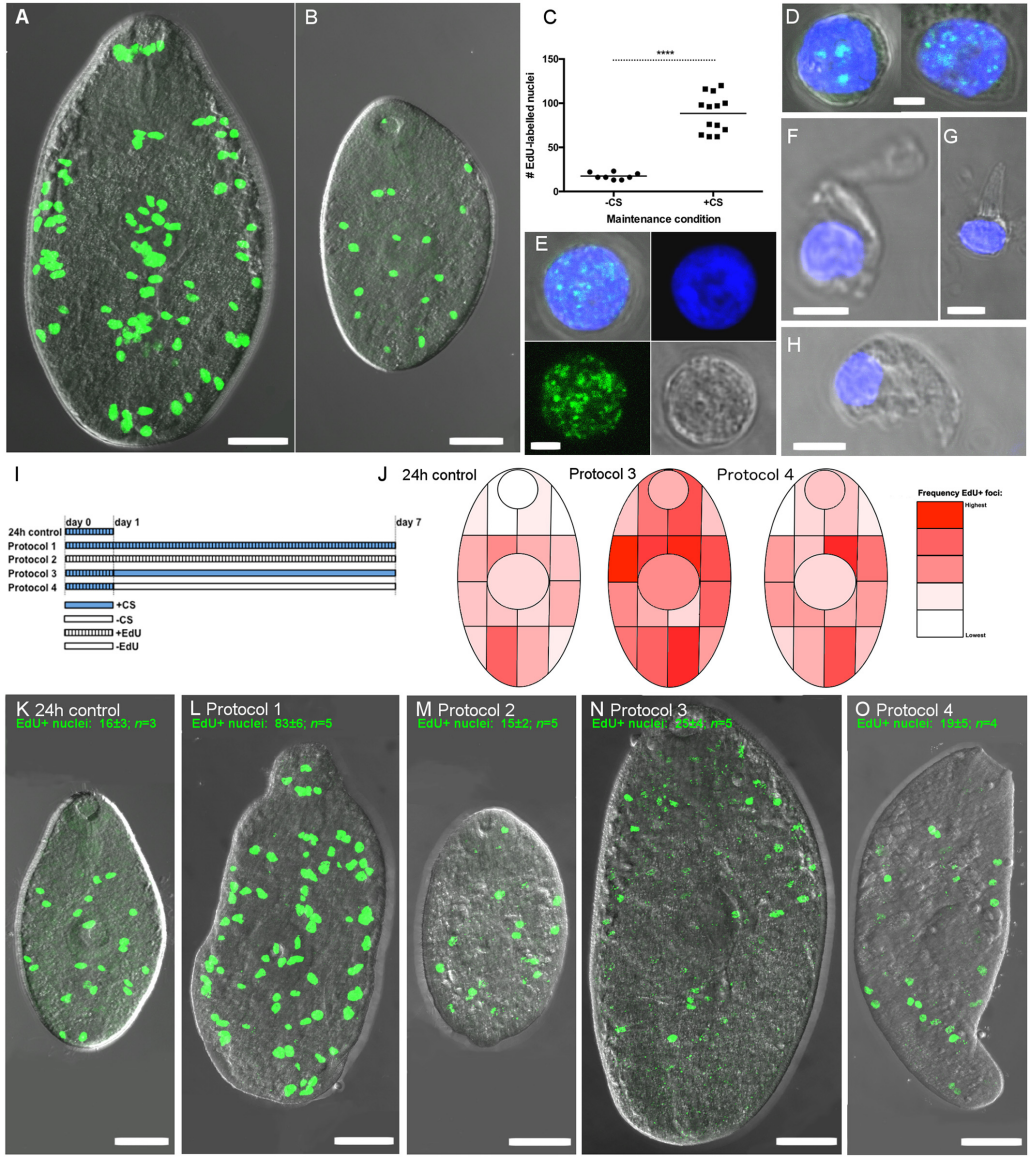
Proliferating cells in growing juvenile *Fasciola hepatica*. Incorporation of 5-ethynyl-2-deoxyuridine (EdU) identifies DNA synthesis occurring during proliferation of cells with neoblast-like morphology. Green fluorescence denotes EdU and blue fluorescence denotes Hoechst 3342 labelling of nuclear DNA. A, B—Distribution of EdU labelled nuclei (EdU+) in fluke grown for 7 days in RPMI+50% chicken serum (A), or unsupplemented RPMI (B); C—Quantification of EdU+ nuclei in non-growing (-CS) vs growing (+CS) specimens; D, E—Morphology of dispersed EdU+ cells; D shows two example cells, E shows single cell and individual fluorescence signals (Hoechst 3342, EdU, brightfield, overlaid); F, G, H—Examples of non-proliferating (EdU-) cells showing distinct morphologies associated with differentiated cells; I, Pulse chase protocols; J, Heatmaps illustrating the change in EdU+ localisation associated with pulse-chase exposure, suggesting that EdU+ nuclei migrate towards differentiated tissue; K-O, example staining patterns of juvenile *F*. *hepatica* from each pulse-chase exposure protocol.

**Fig 7:**
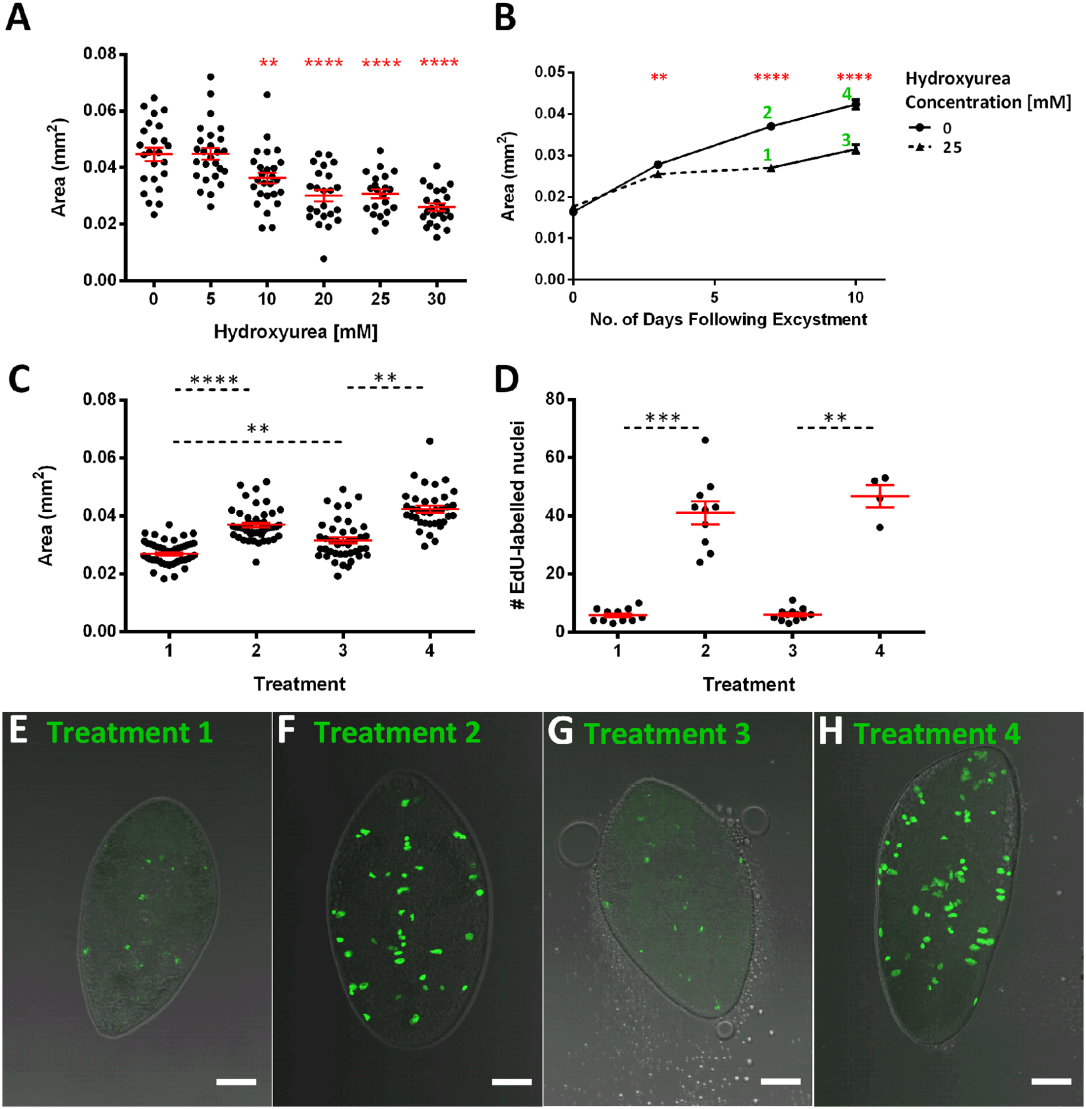
Inhibition of growth with hydroxyurea indicates a role for neoblast-like cells in growth and development. A—Hydroxyurea (HU) produces a concentration-dependent inhibition of worm growth over a 7 day period in RPMI+50% CS; B—7 days HU (25 mM) exposure slows the rate of growth in juveniles maintained in RPMI+50% CS, following HU removal growth rate increases during a subsequent 3 day recovery period. Numbers beside points refer to figures below; C—Increase in worm size following removal of HU (1 vs 3) suggests recovery of growth; D—EdU accumulation does not recover significantly following HU removal. E—EdU labelled nuclei in juveniles maintained in RPMI+50% CS and 25 mM HU over 7 days as seen in ‘Treatment 1’ in B; F—EdU labelled nuclei in juveniles maintained in RPMI+50% CS over 7 days as seen in ‘Treatment 2’ in B; G—EdU labelled nuclei in juveniles maintained in RPMI+50% CS and 25 mM HU for 7 days before HU is removed as seen in ‘Treatment 3’ in B; H—EdU labelled nuclei in juveniles maintained in RPMI+50% CS over 10 days as seen in ‘Treatment 4’ in B; in all graphs mean +/-SEM is presented (shown in red on scatter graphs); in scatter graphs each data-point represents a measurement from an individual worm. Statistical analyses were performed using One Way ANOVA with Dunnett’s post hoc test against untreated “0” sample (A), t-test on days 3, 7 and 10 (B) or Kruskal-Wallis test with Dunn’s post hoc test to compare medians with all other medians (C, D). **, p<0.01; ***, p<0.001; ****, p<0.0001.

We employed two *F*. *hepatica* isolates in this study. All long-term maintenance experiments (Figs 1–5) employed an Oregon strain (Baldwin Aquatics, Oregon, USA). Cell proliferation experiments (Figs 6 and 7) used an Italian strain (Ridgeway Research).

*F*. *hepatica* metacercariae were excysted as described by McVeigh *et al*. [13]. Prior to this excystment method, metacercariae of the Italian strain (supplied on dialysis tubing) were physically popped from their outer wall using a razor blade, before treatment in 10% bleach for 3–5 min. NEJs were held in RPMI before transfer to individual maintenance media as described below.

NEJs were maintained in groups of 10 per well, in round bottomed 96 well plates (Sarstedt) in 250 μl relevant media. All experiments were handled using aseptic technique, and maintained in a humidified, 37°C incubator with 5% CO_2_ atmosphere. Media were changed three times per week, at which time the NEJs were also imaged and their survival rate recorded (viability was assessed by the amount of movement and granulation that was seen in a juvenile; i.e. those juveniles not moving and heavily granulated were considered to be dead). Images were acquired using a Leica MZ125 microscope and attached Unibrain Fire-i digital camera, with measurements of the surface area of a random subset of worms (i.e. those not obscured by other worms) performed using measurement functions within ImageJ software (http://imagej.nih.gov/ij/), and calibrated against a 1 mm scale.

We tested the following media: (i) *Fasciola* saline (FS; Dulbecco’s modified Eagle’s medium (Sigma-Aldrich), supplemented with 2.2 mM Ca[C_2_H_3_O_2_] (Sigma-Aldrich), 2.7 mM MgSO_4_ (Analab), 1 μM serotonin (Sigma-Aldrich), 5 μg/ml gentamycin (Sigma-Aldrich) and 15 mM N-2-hydroxyethylpiperazine-N-2-ethanesulfonic acid (Sigma-Aldrich)); (ii) RPMI 1640 (ThermoFisher Scientific); (iii) NCTC 135 (ThermoFisher Scientific); (iv) PBS (0.15 M NaCl, 0.03 M NaH_2_PO_4_H_2_O (Sigma-Aldrich) and 0.08 M Na_2_HPO_4_ (Sigma-Aldrich) pH 7.4). Each medium was then supplemented with either foetal bovine serum (FBS) (product #A15-151, PAA—The Cell Culture Company), or chicken serum (CS) (product #C5405, Sigma-Aldrich), to varying proportions. All media included an antibiotic-antimycotic solution (Sigma-Aldrich), to final concentrations of 100 U/ml penicillin, 0.1 mg/ml streptomycin and 0.25 μg/ml amphotericin (the latter had no observable impact on worm growth or survival over a 24 h period). Fatty acids (product F7050, Sigma Aldrich), palmitic acid (product P0500, Sigma Aldrich) and BSA (product 05470, Sigma Aldrich) were tested at 0.25 ml/L, 10 μM and 20% w/vol, respectively.

### Preparation of cultured juvenile *F*. *hepatica* for muscle tissue visualisation

Prior to processing flukes for confocal microscopy, worms were paralysed by incubation in 7.14% MgCl_2_ for 1 min at RT. Juveniles cultured for up to 3 weeks post-excystment were free-fixed with rotation in 4% paraformaldehyde (PFA: 4% PFA (Sigma Aldrich) in PBS, pH 7.4) for 4 h at room temperature (RT, 21–25°C). Larger worms (i.e. > 4 weeks after excystment) were flat fixed in 4% paraformaldehyde between microscope slides for 2 h and then free-fixed as described above for a further 2 h, all at RT. Fixed worms were subsequently subjected to 3x 15 min washes in PBSTx (PBS containing 0.5% Triton X-100 (Sigma-Aldrich)) at RT with a final overnight wash at 4°C. Juveniles were then incubated in tetramethylrhodamine isothiocyanate (TRITC)-conjugated phalloidin (Sigma-Aldrich; 200 ng/μl in Antibody Diluent (AbD: PBS containing 0.1% bovine serum albumin (Sigma-Aldrich) and 0.1% Triton X-100 (Sigma-Aldrich)) for 4 h in the dark at RT, before 3x 15 min washes in AbD at RT. Worms were mounted on slides in 8 μl of Vectashield (Vector labs) and viewed under a Leica AOBS SP5 confocal scanning laser microscope.

### Preparation of cultured juvenile *F*. *hepatica* for scanning electron microscopy (SEM)

Juveniles grown in 50% CS in RPMI were fixed in 4% glutaraldehyde (Sigma-Aldrich), for 4 h at 4°C. Following this, juveniles were washed in 0.1 M sodium cacodylate buffer (0.1 M sodium cacodylate (Sigma-Aldrich) buffer (pH 7.4), containing 3% sucrose (Sigma-Aldrich)) overnight (~16 h) at 4°C. Juveniles were then stained in 1% OsO_4_ (90 min, 4°C), followed by three 15 min washes in H_2_O at RT. Juveniles were then washed twice in 70% ethanol for 30 min, twice in 90% ethanol for 20 min and twice in 100% ethanol for 5 min (all at RT). Juveniles were covered with 200 μl hexamethyldisilazane (Sigma-Aldrich) and after 5 min this was removed and 200 μl of fresh hexamethyldisilazane was added and allowed to evaporate overnight (~16 h, RT). Juveniles were then transferred onto stubs and sputter coated for 5 min using a Polaron E5100 Series II before viewing under a FEI Quanta 200 scanning electron microscope. Image J software was used to measure the length of ten spines within the first three rings surrounding the oral suckers of 2–3 juveniles at each week of culture following excystment.

### Preparation of cultured juvenile *F*. *hepatica* for transmission electron microscopy (TEM)

Juveniles processed for TEM were fixed in 4% glutaraldehyde as described above, and then washed in sodium cacodylate buffer at 4°C (60 min). At this point juveniles were cut either longitudinally or transversely before fixation was continued for a further 11 h at 4°C. Juveniles were then washed in 0.1 M sodium cacodylate buffer containing 3% sucrose overnight (~16 h, 4°C) and then stained in 1% OsO_4_ and processed through an ethanol series as described above. At this point juveniles were given two washes in propylene oxide (Agar Scientific) for 5 min at 4°C before embedding in resin (25.2% MNA, 25.2% DDSA, 49.6% agar resin and 1% DMP; Agar Scientific) and propylene oxide in a 1:1 ratio. This was left overnight (~16 h) at RT to allow excess propylene oxide to evaporate. Fresh resin was then placed on the samples and they were left for a further 24 h at RT. Juveniles embedded in resin were polymerised at 60°C for 48 h. Ultrathin sections, 60–70 nm in thickness, were cut on a Reichert Ultracut E ultramicrotome, mounted on bare 200-mesh copper grids, double-stained with alcoholic uranyl acetate (5 min) and aqueous lead citrate (3 min) and viewed in a FEI CM 100 TEM operating at an accelerating voltage of 100 kV.

### Labelling cell division with 5-ethynyl-2-deoxyuridine (EdU)

Visualisation of proliferating cells in *F*. *hepatica* juveniles was achieved by labelling nuclei undergoing DNA synthesis with 5-ethynyl-2-deoxyuridine (EdU; ThermoFisher Scientific). We performed EdU labelling by incorporating 500 μM EdU in the worm maintenance medium (RPMI 1640 +/- 50% CS, as indicated below). EdU exposure of juvenile fluke was performed for 24 h or 7 days, in either +CS or -CS media according to the pulse-chase exposure protocols as follows: (i) 24 h control: 24 h +EdU +CS; (ii) Protocol 1: 7 days +EdU +CS; (iii) Protocol 2: 7 days +EdU–CS; (iv) Protocol 3: 24 h +EdU +CS, 6 days–EdU +CS; (v) Protocol 4: 24 h +EdU +CS, 6 days–EdU–CS. Labelled worms were then processed for detection of EdU-labelled nuclei using the Click-iT EdU Alexa Fluor 488 Imaging Kit (ThermoFisher Scientific), including labelling of nuclear DNA with Hoechst 3342. Our only variation from the manufacturer’s labelling instructions was to fix the worms in 4% paraformaldehyde in PBS for 4 h at RT.

To visualise the morphology of individual labelled cells, we adapted the cell dispersal method described by Collins *et al*. [28]. NEJs incubated overnight in 50% CS containing 500 μM EdU (37°C) were dissociated by incubation in 3.5x Trypsin EDTA in RPMI 1640, for 3 h, with occasional vigorous agitation through a 100 μl pipette tip. Isolated cells were strained by sequential passage through 100 μm and 40 μm cell strainers (Corning), pelleted (200 *g*, 5 min), and resuspended in 4% PFA/PBS in which they were fixed for 30 min. This cell solution was then spotted onto Superfrost slides (ThermoFisher Scientific) and dried at 37°C for approx 15 min. EdU detection was then performed following manufacturer’s instructions, as described above. Samples were mounted on slides in Vectashield (Vector Laboratories), and viewed on a Leica TCS SP5 confocal microscope.

To assess the link between active cell division and EdU labelling, we incubated worms in 50% CS in RPMI containing 500 μM EdU and up to 30 mM hydroxyurea (HU; stocks prepared in H_2_O) for 7 days. In these experiments, HU-containing media were replaced daily because of the instability of HU in solution. In recovery experiments, we incubated juveniles in 50% CS in RPMI containing 500 μM EdU and 25 mM HU for 7 days, followed by removal of HU for a 3 day recovery period.

### Confocal image analysis

EdU labelling was imaged on a Leica SP5 confocal microscope. Whole worms were imaged as maximally projected z-stacks, each generated from 15 optical sections gathered between dorsal and ventral surfaces. To quantify proliferation, we counted the EdU-labelled nuclei in our images using a cell counter plugin for ImageJ. Confocal figures were generated in GIMP (www.gimp.org).

### Collection and analysis of excretory/secretory proteins (E/S) from cultured juveniles

Juveniles grown for 29 weeks in RPMI containing 20%, 50% or 100% CS were washed five times with 250 μl of fresh RPMI at 37°C. Live worms were selected and then collectively incubated (1 worm from 20% CS, 17 worms from 50% CS and 4 worms from 100% CS) for 4 h in RPMI without CS. For proteomic analyses, supernatants containing each E/S sample were pooled and lyophilised before being reconstituted in 50 μL MilliQ H_2_O, with 5% of each sample analysed by 1DE (4–20% Mini-PROTEAN TGX Gel Biorad) using SilverQuest Silver staining kit (Invitrogen). The remaining samples were subjected to MS/MS analysis using an Orbitrap Elite 1410 Mass Spectrometer (Thermo Scientific) at the LaTrobe Institute for Molecular Sciences (LIMS) Proteomic Mass Spectrometry Facility. Proteins were identified using an in-house *F*. *hepatica* transcriptome database and by performing a BLASTp of the NCBI and GenBank databases to reveal associated accession numbers.

### Statistical analysis

All graphs were produced and statistical tests were carried out in GraphPad Prism 6 for Windows (GraphPad Software, La Jolla California USA, www.graphpad.com). In cases where sample variances were equal (determined by F test or Brown-Forsythe test) parametric tests were used (t test and ANOVA) but when variances were not equal non parametric tests were used (Mann Whitney U-test and Kruskal-Wallis). Post-hoc tests were used to compare means/medians of multiple groups and were chosen based on whether comparisons were needed between all groups or against a control group.

## Results

### Chicken serum promotes extended survival of juvenile *F*. *hepatica in vitro*

In parallel to our attempts to develop RNAi methods for *Fasciola* spp. [11,13], we have also made efforts to improve *in vitro* maintenance methods to enable long-term study of phenotypic changes following RNAi or drug treatments. From initial experiments employing a DMEM-based “*Fasciola* saline” (FS), performed over less than 24 h [11], we subsequently reported that unsupplemented RPMI enabled *in vitro* study of viable juvenile fluke for up to 3 weeks [13]. Here, we tested both of these media as well as NCTC 135, in the presence/absence of varying levels of foetal bovine serum (FBS) and chicken serum (CS) (S1 Fig and S1 Table).

Early experiments using un-supplemented base media illustrated that FS and NCTC were a much less effective maintenance media than RPMI (50% survival time: FS, 9 days; NCTC, 9 days; RPMI 26 days), leading us to disregard FS and NCTC from further experiments. Success of previous studies in using RPMI as a medium for schistosome maintenance [29], alongside the significantly lower purchase cost of RPMI, led us to focus on RPMI in remaining experiments.

Supplementation of RPMI with FBS did not improve worm survival, and in fact seemed to impede longevity (50% survival time: RPMI, 26 days; +5% FBS, 23 days; +10% FBS, 22 days; +20% FBS 19 days; +50% FBS, 14 days; 100% FBS, 15 days; S1 Fig). At this stage, we tested chicken serum (CS), in line with a previous study [23] and found that RPMI supplemented with CS was an extremely effective promoter of fluke longevity. Fig 1 shows that 50% CS in RPMI supported 65% juvenile survival after 29 weeks (at which time the experiment was purposely terminated). We found 50% to be the optimal concentration of those we tested, since 100% CS was less effective (50% survival at 4 weeks; 13% survival at 29 weeks), with dilutions below 50% CS displaying concentration-dependent impacts (50% survival: +5% CS, 4 weeks; +10% CS, 5 weeks; +20% CS, 8 weeks). Note that one juvenile in 20% CS in RPMI did survive to the end of the trial but was considered an outlier in terms of both survival and growth (see below). All of these parameters represent significant improvements on fluke survival in RPMI±FBS, and to our knowledge represent the longest reported periods of *F*. *hepatica* maintenance *in vitro*. In additional experiments not detailed here, we have since achieved maintenance/growth of fluke for 13 months using these methods. Finally, we tested whether supplementation of RPMI with a fatty acid mixture, palmitic acid or bovine serum albumin would stimulate fluke growth but no effect was observed relative to the growth observed with RPMI+50% CS (S2 Fig).

### Chicken serum promotes growth and development of adult-like morphology in juvenile *F*. *hepatica* maintained *in vitro*

In addition to the improved survival imparted by CS supplemented RPMI, we also noted more rapid growth of fluke compared to those maintained in FBS. Fig 1B illustrates that even after 1 week juveniles maintained in RPMI containing either 50% or 100% CS were significantly larger than those in any other media (mean Week 1 area: RPMI, 0.02 μm^2^; 50% CS in RPMI, 0.04 μm^2^; 100% CS, 0.05 μm^2^) (Week 1 Kruskal-Wallis: H = 143.9, 241 d.f., P < 0.0001; S2B Table). By 29 weeks post-excystment, juveniles maintained in 50% CS in RPMI had grown to 38.5x their original size (worm area: week 0: 0.02 mm^2^; week 29: 0.77 mm^2^), although there was no significant growth after 20 weeks. Notably, we observed a considerable increase in the variability of worm sizes at later time points (distance between 95% confidence intervals: 0 weeks, 0.00137 μm^2^; 10 weeks, 0.1328 μm^2^; 20 weeks, 0.4381 μm^2^; 29 weeks, 0.5519 μm^2^), suggesting differences in the growth capacities of individual worms.

In growing worms, we used confocal microscopy to observe increased gut complexity and the development of reproductive tissue, both of which are indicative of transit towards more adult-like characteristics. NEJs and early (up to a week post excystment) *in vitro* cultured juvenile fluke exhibited simple digestive caeca and no reproductive structures. From two weeks onwards, both branched digestive caecae and uterine tubing were visible in the region posterior to the acetabulum (Fig 2A and 2B). By 2 to 3 weeks post-excystment all juveniles exhibited primary branching of digestive caeca (Fig 2A and 2D), with 66% of fluke developing uterine tubing posterior to the ventral sucker (Fig 2B, 2D and 2E). We consider this tubing uterine in nature because it is consistent with the location of the uterus in mature *F*. *hepatica* [30], and is therefore indicative of developing female reproductive structures. At 4 weeks post excystment, secondary gut branches had appeared in 55% of fluke (Fig 2A and 2F).

After 29 weeks maintenance *in vitro*, all surviving juveniles had undergone considerable growth (Fig 1B) with all individuals exhibiting secondary branching of the digestive caeca (Fig 2A) and uterine tubing posterior to the acetabulum (Fig 2B). Due to the larger size of these worms, we were able to view the latter structures in greater detail, observing an extended uterus (Fig 2G).

An extreme example of the variable growth capacities of individual worms was exhibited by a single fluke, maintained in 20% chicken serum for 29 weeks. This fluke grew larger than any other in this study. Although an outlier in terms of growth rate, it was nonetheless instructive in terms of the potential for *in vitro* development in this system. Specifically, this individual grew to 239x its original size, reaching 4.3 μm^2^ in area and 3 mm in length (Fig 3A), whilst juveniles in 50% CS in RPMI reached ~38.5x their original size (0.77 μm^2^) with a mean length of 1.45 mm. This fluke had distinctive external morphology more representative of an adult than a juvenile fluke, characterised by: (i) a more anterior position for the acetabulum relative to the central positioning seen in juveniles (Fig 2C); (ii) development of an oral cone and ‘shoulders’, with a leaf-like shape, distinct from the more vermiform juvenile; and, (iii) the loss of the probing movement displayed by migrating stage fluke and the adoption of a wave–like movement characteristic of adults (S1,S2 Movies). This specimen had the most well developed gut of any observed in this study, with distinct tertiary branches of the caecae (Fig 3B). In addition to considerably longer and more elaborate uterine tubing, this worm also displayed a putative ootype (Fig 3B). These structures were connected to the gonopore via a gonoduct, tracking dorsally to the acetabulum (Fig 3B and S3 Fig). We did not observe testes *per se*, but this specimen was unique amongst our samples in exhibiting tubing putatively associated with male gonads in the anterior region where testes exist in adult worms (Fig 3B).

### *In vitro* development includes ultrastructural changes to the fluke tegument

In addition to the gross internal developmental changes described above, our maintenance method also triggered changes to the ultrastructure of the inner and outer tegument that signify development of juvenile fluke towards more immature- and adult-like forms. We detected these changes using both SEM and TEM. SEM observation of the fluke surface revealed that tegumental spines grew in length significantly over the course of the study (mean spine length around oral cone: 0 weeks, 0.52 μm; 2 weeks, 1.37 μm; 4 weeks, 2.26 μm; 29 weeks, 3.28 μm) (Fig 4A; Kruskal-Wallis: H = 134, 157 d.f., P < 0.0001; S3 Table). Tegumental spines also changed in morphology during development; Fig 4B illustrates the progression from the short, stunted spines of the NEJ, towards the more distinct, regular arrangement of spines as rings circling the fluke visible at 2 weeks post-excystment (Fig 4C). Similarly, NEJs lacked spines on the posterior ventral surface, developing spines in this region by 2 weeks post excystment. This occurred at an even slower rate on the posterior dorsal surface, where spines were visible only after 29 weeks post excystment (S4 Fig). By 4 weeks post excystment spines began to develop multiple tips (Fig 4D). This process was first visible in spines on the ventral anterior surface, which at this point had two tips (Fig 4D). By 29 weeks post excystment we observed spines with two, three or four tips (Fig 4E) compared to the 8-tipped spines seen in *ex vivo* adult fluke.

The development of cultured juveniles in our maintenance medium also triggered changes in the internal ultrastructure of the fluke tegument, most evident from the increase in depth of the tegument syncytial layer to ~1800 nm (29 weeks) (Fig 5A; Kruskal-Wallis: H = 38.07, 45 d.f., P < 0.0001; S4A Table), which is comparable to the tegument depth observed in immature fluke recovered from the liver of mice 1–2 weeks post-infection [31]. In addition, a significant increase in the depth of surface invaginations (tegumental in-folds) was observed over the weeks following excystment (mean invagination length: 0 weeks, 218.1 nm; 2 weeks, 308.4 nm; 4 weeks, 1053 nm; 29 weeks, 2146 nm) (Fig 5B; Kruskal Wallis: H = 46.82, 84 d.f., P < 0.0001; S4B Table).

Beyond these gross changes, we also observed differences in the organellar composition of the tegumental syncytia over time. Fig 5C shows that the NEJ tegument is barely discernible from underlying tissues. However, by 1 week post-excystment there had been a rapid change in the tegument ultrastructure, with the development of a clear syncytium and the appearance of tegument-specific secretory vesicles: T0 (large, spherical electron dense structures) and T2 bodies (biconcave discoid structures with electron lucent contents) (Fig 5D). This process remained visible at 2 weeks post-excystment but with the additional presence of T1-like bodies (i.e. vesicles containing both electron-dense and–light regions; Fig 5E and 5F) which are known to appear in immature flukes in the liver [31]. By 4 weeks post-excystment the tegument appeared devoid of T0 bodies, with the T1-like bodies having developed into mature T1 bodies with their typical ‘cartwheel’ appearance (Fig 5G). By 29 weeks post-excystment, T1 bodies were tightly packed in the syncytium with evidence of T2 bodies also present (Fig 5H).

### Neoblast-like cells proliferate in fluke maintained *in vitro*

To visualise patterns of cell division associated with fluke growth and development, we labelled worms with EdU, enabling the detection of cells undergoing active DNA synthesis. Fig 6A and 6B show that EdU-labelled (EdU+) nuclei accumulated at different rates in growing (+CS) and non-growing (-CS) worms when incubated continuously in EdU for 7 days. EdU accumulation over this period was higher in growing than non-growing fluke (-CS +EdU # nuclei 17±1, *n =* 8; +CS +EdU # nuclei 88±6, *n* = 13; p<0.0001; Fig 6C), suggesting that proliferating EdU+ cells directly contribute to fluke growth by increasing the total cell count. The spatial pattern of EdU+ accumulation in growing worms is notable: in non-growing worms, EdU+ nuclei were distributed throughout the parenchyma, but appeared absent from the anterior 1/3 of the worm (Fig 6B). In growing 7 day old fluke, EdU+ nuclei accumulated in a distinct pattern of three clusters of nuclei in (i) the oral sucker, (ii) the anterior-posterior midline and (iii) the lateral margins (Fig 6A). Only in +CS worms did we observe EdU+ nuclei in the anterior 1/3 of the fluke. The appearance in growing worms of EdU+ cells in locations not labelled in non-growing worms, suggests expansion and movement of these cells into new areas, and probably their differentiation in those areas. Taken alongside the increased expression of typical neoblast marker genes (argonaut and nanos) in growing compared to non-growing worms (S5 Fig) [32], we consider that the EdU+ cells of *F*. *hepatica* juveniles resemble flatworm neoblasts. To examine the morphology of EdU+ cells, we performed tryptic digests of whole juveniles labelled *in vitro* for 18h (+CS+EdU), with EdU detection and confocal analysis of dispersed cells fixed onto microscope slides. All of the EdU+ cells we detected displayed the gross morphology characteristic of neoblasts as described in other flatworms—relatively small, rounded cells with a prominent nucleolus, and scant cytoplasm surrounding the nucleus (Fig 6D and 6E). Some EdU- cells also had a similar appearance, but most of these had distinctive morphologies suggestive of differentiation (Fig 6F–6H).

In non-growing worms, while EdU+ nuclei are visible, accumulation of EdU labelling is not significantly different between worms incubated in EdU for 24h or 7 days (24h EdU+ nuclei 16±3, *n* = 3; 7 day EdU+ nuclei 17±1, *n* = 8; Fig 6B and 6M); neither does the localisation of EdU+ nuclei change between these time points in non-growing samples (Fig 6B and 6M). This suggests that in addition to the population of proliferating cells that contribute directly to worm growth, presumably through expansion and differentiation, there is a sub-population of EdU+ cells that proliferate, possibly for maintenance or self-renewal, but which progress towards differentiation only upon exposure to an appropriate developmental signal. Following inhibition of DNA synthesis and neoblast proliferation by exposure to HU (7 days; see below), we tested the self-renewal potential of neoblasts after the removal of HU by following EdU re-accumulation in worms over a 3 day recovery period. Although we observed the significant recovery of growth in these worms, we did not detect expansion of the few remaining neoblasts following this recovery period. It is possible that the 25 mM HU that we employed triggered cell death in our neoblast population [33]; future work should titrate HU to the lowest possible concentration for use in renewal experiments.

Neoblasts are presumed pluripotent when they originate, only subsequently differentiating into mature cell types within specific tissue or organ systems (although there is evidence for several sub-populations with potentially distinct fates amongst planarian neoblasts [34]). This developmental process requires neoblasts to migrate from their origins in the parenchyma to their mature site of residence in a differentiated tissue. To investigate whether *F*. *hepatica* EdU+ cells behaved similarly, we performed pulse-chase experiments with the aim of determining the spatial fate of EdU+ cells labelled in a 24h pulse, following a 6 day chase period (protocols described in Fig 6I). Both growing and non-growing pulse-chase worms displayed a spatial shift in EdU+ localisation compared to 24h controls (Fig 6J, 6K, 6N and 6O), including movement into previously unlabelled areas, consistent with previous reports of flatworm neoblasts [35, 36].

### Neoblast proliferation supports growth of juvenile fluke cultured *in vitro*

To further examine the link between neoblasts and worm growth, we examined the impact of inhibiting DNA synthesis (using hydroxyurea, HU) on neoblast proliferation and worm growth rate. Fig 7A illustrates that HU has a concentration-dependent impact on growth of the juvenile flukes over a 7 day observation period, with statistically significant inhibition of growth vs untreated controls at ≥10 mM (worm area: 0 mM HU, 0.045±0.002 mm^2^, n = 24; 25 mM HU, 0.03±0.001 mm^2^, n = 26, p<0.0001). HU simultaneously inhibits EdU+ proliferation, such that 10- fold fewer EdU+ nuclei are visible in worms treated with 25 mM HU than in untreated controls over a 7 day incubation (Fig 7D, 7E and 7F; Mean±SEM # EdU+ nuclei/worm: 0 mM HU, 41±3.9; 25 mM HU, 5.8±0.7; Mann Whitney U test, n = 21 p<0.0001). Upon removal of HU, worm growth rate increases (Fig 7B) with a significant increase in size over the following 3 days (Fig 7C; Mean±SEM worm area: Day 7, 0.026±0.0005; Day 10, 0.031±0.001; Mann Whitney U test, n = 97, p<0.001). However, this significant increase in growth does not correlate with a significant increase in the number of EdU-labelled nuclei (Fig 7D and 7G). The correlation between worm growth and neoblast proliferation is consistent with neoblasts representing the major source of cellular proliferation in support of worm growth and development.

### Proteomic analysis of E/S proteins from *in vitro* maintained fluke

We performed MS/MS analysis of E/S samples from juveniles maintained for 29 weeks in either 20% CS in RPMI (n = 1 fluke), 50% CS in RPMI (n = 17 fluke) or 100% CS (n = 4 fluke). Our aim was to compare the protein content of E/S gathered from *in vitro* maintained juveniles with the previously described distinct E/S profiles of NEJ, immature and adult *F*. *hepatica*. We hypothesised that this would provide proteomic evidence of fluke development. We identified peptides from 61 different *F*. *hepatica* proteins, with distinct profiles in each of our juvenile groups: 17 proteins in the juvenile grown in 20% CS in RPMI, 52 proteins in those juveniles grown in 50% CS in RPMI and 27 proteins identified in the juveniles from 100% CS. Within these juvenile profiles we discovered ten proteins known to be solely expressed in the E/S of immature and/or adult fluke, including cathepsin L 1A, L2 and L5, cathepsin B2 and B7, Legumain 1 and 5, a prolyl-carboxypeptidase, Thioredoxin H type 1 and GST sigma 1 (Table 1; [25,27]). Since our *in vitro* juvenile E/S profiles more closely resemble the known profile of immature/adult worms than NEJs, we consider that our *in vitro* maintenance system enables study of the temporal development of E/S output in *F*. *hepatica*.

**Table 1:** Excretory/secretory proteins of *F*. *hepatica* from fluke grown in 20% or 50% CS in RPMI or 100% CS for 29 weeks *in vitro*.

Annotation	NCBI/ Genbank Accession no.	Annotation^1^^,^^2^	% AA identity	20%	50%	100%	NEJ	Immature	Adult
				CS	CS	CS			
**CL1A**	Q24940.1	Q24940^1^	97	**+**	+	+	-*	+*	+*
**CL2**	ABQ95351.1	A5Z1V3^1^	99	**+**	+	+	-*	+*	+*
**CL3**	ABW24657.1	A8W638^1^	100	**+**	+	+	+*^#^	+*	-*^##^
		DQ534446^2^							
**CL5**	AAF76330.1	Q9NB30^1^	95	**+**	+	-	-*	+*	+*
**Cathepsin B2**		Q817B2^1^	99	-	+	-	-*	+*	-*
**CB3**	CAO98753.1	DQ534445^2^	97	-	+	+	+^#^	n/a	-^##^
**Putative cathepsin B7**		Fhep44e10.q1k^1^	98	-	+	+	-*	-*	+**
**Legumain-like precursor 1**	CAC85636	Q711M2^1^	95	-	+	+	+*	+*	-*
**Legumain 1**	ABQ02437.1	A6Y9U8^1^	99	-	+	+	-*	+*	+**
**Putative legumain 2**		Fhep29h09.q1k^1^	100	**+**	+	+	+*	-*	-*
**Putative legumain 5**		Fhep21f02.q1k^1^	97	-	+	-	-*	-*	+**
**Putative prolylcarboxy- peptidase (s28)**		Fhep30b01.q1k^1^	99	-	+	+	-*	+*	+*
**Thioredoxin H-type 1**	Q9U1G7	Q9U1G7^1^	100	+	-	-	-*	-*	+*
**GST-Sigma 1**	2WB9	Q06A71^1^	97	+	+	-	-*	+*	-*

Proteins observed are annotated based on the percentage (%) of amino acid (AA) identity to previously described sequences (NCBI/ GenBank, UniProtKB).

^1^Robinson et al. [27] annotation;

*Robinson et al. [27] (MS/MS data);

**Robinson et al. [27] (transcript data);

^2^Cancela et al. [25] annotation;

^#^Cancela et al. [25] (MS/MS data);

^##^ Cancela et al. [25] (transcript data);

n/a: not available

## Discussion

While recent progress has been made in the development of sequence datasets and functional genomics tools for liver fluke, *in vitro* maintenance methods to support the application of these resources for the study of *F*. *hepatica* biology are lacking. In an attempt to address this we have developed a simple method for the *in vitro* maintenance of *F*. *hepatica* juveniles. Using these methods to study growth and developmental processes, we show here that: (i) our *in vitro* system recapitulates aspects of known morphological and behavioural indicators of *F*. *hepatica* development towards adulthood; (ii) *F*. *hepatica* growth and development is likely supported by the proliferation of somatic neoblast-like cells; and, (iii) the proteomic profile of E/S material released by the cultured flukes changes to a more immature/adult fluke–like profile providing evidence that significant fluke development is occurring.

This work originated from our attempts to improve the survival of juvenile fluke during RNAi experiments, where our aim was to maintain fluke for a period long enough to detect phenotypic changes following transcriptional silencing. The primary finding here was that although worms would grow and survive for up to 3 weeks in FBS, the survival and growth rate of juvenile worms was vastly improved by substituting chicken serum (CS) for FBS. CS has previously been used in the cultivation of *Maritrema novaezealandensis*, a trematode parasite of Red-Billed Gulls in New Zealand [37] as well as for the culture of *Philophthalmus sp*. and *Gynaecotyla adunca* which both have avian definitive hosts [38,39], so its use in helminth culture media is not without precedent. Why CS represents such an improvement on FBS, a product of *F*. *hepatica*’s definitive host, remains unclear. In addition to likely differences in the complements of growth hormones/promoters between the two sera that would impact on their relative capacities to stimulate growth, there exists a previously reported compositional difference, where CS is known to contain higher levels of triglycerides and cholesterols [40] than FBS. This is an intriguing factor given that fluke are thought to lack the capacity to synthesise fatty acids *de novo* [41] so it is possible that the higher lipid content of CS may reflect its greater capacity to support growth.

Attempting to improve upon earlier studies in this area [23,24] we quantified 2 dimensional area (rather than length) as a measure of worm growth, reasoning that this measure would be less susceptible to fluctuation caused by the considerable changes in length that occur during normal motility in juvenile fluke. If we do compare lengths between juveniles in this study and previous work, the largest juvenile in our trials reached 3 mm in length, similar to the 3.1 mm fluke obtained after 14 weeks fluke culture by Smith and Clegg (1981) using 50% human sera in RPMI with 2% human red blood cells. However, in that study, survival was less than 50% at 14 weeks and only 96 of the 240 total juveniles were measured (specifically, the six fastest growing juveniles in each group) [24]. Additionally, these authors reported that seven flukes grew to 6–7 mm after 14 weeks. The data presented in our study encompass the mean growth of all fluke (across two independent trials; note that in two other trials in which fluke growth was not monitored throughout, worms had reached sizes similar to those reported here after 6 months), thereby representing a more complete overview of the growth platform. More significantly, fluke survival was greater in our trials with 65% survival after 29 weeks.

Growing juveniles exhibited considerable variation in growth capacity, as exhibited by the range of sizes seen, particularly during the latter stages of the study. These differences may be due to their relative abilities to respond to chemical growth promoting signals in medium, to extract adequate nutrition from the medium, or to physical differences in their cellular capacities for growth and differentiation. Regardless, it is worth noting that the flukes used here in long-term maintenance experiments originated from a wild type population of metacercariae obtained from the USA, the genetic diversity of which may be responsible for the range of growth phenotypes reported.

Comparison to *in vivo* juveniles, which in mice grow to an average of 1 mm in length after 8–9 days [30] and 3 mm after 15–20 days in the liver, shows that growth clearly occurs more slowly in our *in vitro* system than is the case *in vivo*. These comparisons suggest that our *in vitro* fluke maintenance system facilitates the development of fluke comparable to immature liver stage *in vivo* parasites. To determine whether morphological measures support this conclusion, we employed confocal and electron microscopy to examine changes to gut, reproductive and tegument tissues. Interestingly, the *in vitro* development of caecal branching in the juveniles corresponded with that of size-matched *in vivo* juveniles; *in vitro* juveniles of ~0.5 mm in length (3 weeks growth post-excystment), 1.45 mm (29 weeks growth) and 3 mm (29 weeks growth, 20% CS outlier), respectively, exhibited primary, secondary and tertiary caecal branching, as did their size-matched *in vivo* relatives [30]. These observations indicate that development of the digestive tract is linked to worm size, rather than to developmental age.

A similar observation was made regarding the development of the reproductive system. *In vitro* juveniles of ~0.5 mm in length (3 weeks post-excystment) exhibited some development of uterine tubing, of a similar extent seen in size matched (1 week post infection) *in vivo* parasites [30]. However, where *in vivo* parasites possessed a visible gonoduct and testes at this stage, our *in vitro* juveniles did not. By 29 weeks, our 1.45 mm *in vitro* juveniles had fallen considerably behind the development of 1.5 mm *in vivo* parasites, which have a well-developed gonoduct and significant oviduct, ovary and testes development [30]. None of these features were visible in our 1.45 mm *in vitro* juveniles; even though our 3 mm outlier fluke exhibited much more reproductive tissue development than any other fluke in this study, gonads were not detected. This individual displayed extensive uterine development, including the complex folds noted for this structure *in vivo* [30]. There was also development of the gonoduct, anterior to the ventral sucker, although a gonopore was not visible. This specimen also possessed tube-like structures in the region of the testes, potentially representing the vas deferens leading from the testes to the ootype. Although our *in vitro* fluke can be considered stunted versions of comparable *in vivo* parasites, our juveniles do compare favourably with the development achieved *in vitro* in previous studies, where uterine development was reported in 6–7 mm juveniles, albeit in the absence of ovarian development or evidence of eggs [24]. We did not observe development of a cirrus, consistent with previous reports [23,24].

Previous *in vitro* culture studies did not examine tegumental development. Our SEM analyses revealed that development of the tegumental surface under our *in vitro* assay system largely resembles that seen *in vivo*. In contrast to the rather poorly developed spines of NEJs [42], we observed growth and the increased distribution of spines across the surface of fluke growing *in vitro*. After one week of growth *in vitro* spines were present across the whole ventral surface and showed a significant increase in length as well as an increased density, especially on the posterior dorsal surface. This growth-related increase in spine density has been observed previously in fluke recovered from murine hosts [43]. During the subsequent three weeks of growth *in vitro* we observed no further increases in tegumental spine distribution although, after 29 weeks, spines were found across essentially the entirety of the fluke surface, including the dorsal, posterior surface from which they had been absent at earlier time points.

We also noted morphological changes to the spines, characterised by the development from single-tipped to multi-tipped spines in later stages. We first noted multi-tipped spines at 4 weeks post-excystment, firstly on the anterior ventral spines where secondary tips began to appear. This change occurs at around 2 weeks post infection *in vivo*, although after 3 weeks *in vivo* spines had as many as 8-points [43]. Even after 29 weeks *in vitro*, our juveniles in 50% CS in RPMI had spines bearing a maximum of 3–4 tips, again suggesting that although our system triggers *in vivo*-like developmental changes, these changes occur at a slower rate *in vitro* than *in vivo*.

The subsurface ultrastructure of the tegument also showed changes in line with development in our growing fluke. Developmental changes to the composition and diversity of various organelles and structures in the tegumental syncytium have been described in some detail in *F*. *hepatica* [31]. One of the most well studied aspects of this process is the dynamics of the secretory vesicles (termed T0, T1, T2 bodies [31]) contributing to tegumental turnover and renewal. In this process, T0 bodies are replaced by T1 bodies as the fluke develops towards adulthood, while there is a concurrent increase in the number of T2 bodies [31]. These bodies are thought to play a key role in tegument development and defence from the host immune system as the juvenile fluke migrates through the liver parenchyma. TEM studies of our *in vitro* fluke revealed that T0 to T1 transformation occurs *in vitro* over a similar timescale to that seen *in vivo*. Juveniles cultured *in vitro* in our trials exhibited T0 and T2 bodies after 1-week post-excystment with T1 bodies appearing after 2 weeks. T1 bodies began to outnumber the T0 bodies after 3 weeks of growth, with only T1 and T2 bodies visible by four weeks post-excystment. *In vivo*, the process occurs only slightly faster, with T1 bodies first appearing after 5 days of infection in mice and representing the most numerous tegumental body after 2 weeks when the fluke are in the liver parenchyma. This suggests that tegument development is timed from excystment, rather than being related to fluke growth/size. The appearance of T2 bodies provides further evidence as to the development of these juveniles as it again suggests that the ultrastructural development of these bodies is progressing in a manner comparable to that seen *in vivo*. T2 bodies appear *in vivo* after two days of infection in mice (in syncytial cell bodies with only a few at the surface of the tegument syncytium) but only predominate in mature, adult fluke [31].

To investigate the cellular basis of the growth/development phenotypes described here, we used confocal microscopy to examine the nature of proliferating cells in juvenile *F*. *hepatica*. Given that neoblasts (pluripotent stem cells) appear to be the only proliferating somatic cells in other flatworms [32, 35, 44, 45], our hypothesis was that similar progenitor cells would also drive growth and development in *F*. *hepatica*. We tested this hypothesis using a commercially available EdU labelling kit to examine the location, morphology and behaviour of proliferating cells, and here provide the first description of putative neoblasts in *F*. *hepatica*, showing that EdU+ cells in juvenile *F*. *hepatica*: (i) have neoblast-like morphology; (ii) display migratory behaviour consistent with flatworm neoblasts; (iii) support worm growth. By detecting incorporation of EdU, a thymidine analogue, into newly synthesised DNA, we identified strong labelling of cellular nuclei (co-stained with Hoechst 3342) scattered through the posterior two thirds of juvenile fluke. *F*. *hepatica* EdU+ cells arise within the parenchyma below the tegument and muscle layers, and posterior to the cerebral ganglia. These cells show the typical morphology of neoblasts described in other flatworms (round cell, large nucleus, prominent nucleolus, scant cytoplasm). To explicitly link the presence of neoblasts with fluke growth, we first examined the correlation between neoblast numbers and worm growth. Fig 7 shows that in worms maintained in 50% CS in RPMI, alongside worms in unsupplemented RPMI, in the presence of 500 μM EdU for 7 days we saw growth only in 50% CS worms, while the accumulation of EdU+ nuclei (indicating the presence of cell division), was much greater in RPMI plus CS than in RPMI-only worms. In fact, the numbers of EdU+ nuclei did not increase in our non-growing worms during 7 days incubation in RPMI. These data suggest that enhanced neoblast proliferation in CS-containing media contributes to worm growth. To test this link further we used HU (an inhibitor of ribonucleotide reductase, the enzyme that catalyses production of DNA from RNA) to inhibit DNA synthesis in, and subsequent mitosis of, EdU+ cells. At concentrations of 10 mM or greater, HU inhibited worm growth and lowered neoblast abundance in CS-maintained worms. This suggests that neoblast proliferation provides the increased cellular mass that drives worm growth.

Using pulse-chase microscopy, we were able to confirm that the localisation pattern of fluke neoblasts changes over time, suggesting that they migrate away from their site of origin. Following a pulse/chase regime of 1 day pulse, 6 days chase, we detected a distinct shift in spatial localisation of EdU+ labelling in growing worms. Fig 6 illustrates this shift where EdU+ nuclei in “pulsed” worms were seen across the core and lateral margins, but only within the posterior two thirds of the worms. This pattern changed in “chased” worms where EdU+ nuclei were concentrated around the flanks, including within the anterior one third of the flukes. We hypothesise that these may represent differentiated tegumental nuclei as described in *S*. *mansoni* [28]. These data represent the first description of neoblast-like cells in *F*. *hepatica*, and will support further investigations into the molecular genetics and functional genomics of these pluripotent cells. Such studies will provide further biological insight into the intriguing stem cell system of parasitic flatworms, and may highlight developmental targets for future control interventions.

Lastly, proteomic analysis of E/S proteins provides further insight into the maturation of the *in vitro* cultured fluke. The presence of the cathepsins CL1A and CL2 in all three E/S samples, and CL5 in two of the E/S samples, clearly indicates that the *in vitro* cultured fluke have developed significantly from the juvenile stage with the supplementation of 20–100% CS. CL1, CL2, and CL5 are the main proteases known to be produced by adult *F*. *hepatica* [46,47]. Other proteins known to be produced by the immature and/or adult flukes were also identified, such as CL3, CB2, putative cathepsin B7, Legumain 1, putative prolylcarboxypeptidase, Thioredoxin H-type 1 and GST-Sigma 1 [25,27]. The E/S protein profile of flukes grown in 50% CS in RPMI exhibited the closest similarity to the known profile of immature/adult E/S. The protein profiles observed indicate that the flukes had not fully switched from the juvenile stage as two proteins that are markers for juvenile E/S (CB3 and putative legumain 2) were observed in the *in vitro* developed worms. It is possible that the variation in E/S profile observed in our *in vitro* fluke, relative to that observed from flukes recovered from animals, may be due to the lack of host cues required for complete maturation. Further careful manipulation of the culture conditions may allow the production of flukes that better mimic those recovered *in vivo*.

In summary, this study describes a set of simple methods enabling long-term *in vitro* maintenance of *F*. *hepatica* juveniles, which also permit constant monitoring of development, survival, growth and other phenotypic measures in maintained fluke. We have used this system to: (i) profile developmental changes in several fluke tissues that resemble those processes reported in *in vivo* parasites; and, (ii) provide the first description of neoblast like putative stem cells in *F*. *hepatica*, implicating these cells as essential for fluke growth and development. These methods will therefore support the development of *in vitro* assays for flukicidal drug and vaccine target validation screens, including the use of functional genomics tools such as RNAi [11–14]. Given that our *in vitro* methods recapitulate several aspects of fluke development *in vivo*, albeit at a slower rate, our system also has clear potential to reduce animal use that is currently unavoidable for the production of late juvenile or early adult parasites.

## Supporting Information

S1 Table Statistical data on juvenile *Fasciola hepatica* growth *in vitro*. Fluke were maintained in various base maintenance media and with supplementation with Foetal Bovine Serum (FBS). A—ANOVAs and Kruskal-Wallis tests with associated post hoc tests, revealing different survival rates of juveniles in different base media and at various FBS percentages; B—Kruskal-Wallis tests with associated post hoc tests and Mann Whitney U test revealing different growth rates of juveniles in different base media and at various FBS percentages.
(XLSX)

S2 Table Statistical data on juvenile *Fasciola hepatica* growth *in vitro* in RPMI with Chicken Serum (CS) supplementation. A—ANOVAs and associated post hoc tests revealing different survival rates of juveniles in different percentages of CS; B—ANOVA and Kruskal-Wallis tests with associated post hoc tests revealing different growth rates of juveniles in different percentages of CS.
(XLSX)

S3 Table Statistical data on tegument spine length differences in juvenile *Fasciola hepatica* maintained *in vitro*. Data include Kruskal-Wallis tests and Dunn’s post hoc tests on fluke grown in 50% Chicken Serum in RPMI *in vitro*.
(XLSX)

S4 Table Statistical data on juvenile *Fasciola hepatica* tegument development during maintenance *in vitro*. Fluke were grown in 50% Chicken Serum in RPMI. A—Kruskal-Wallis and Dunn’s post hoc test revealing significant differences in the thickness of the syncytium over the weeks following excystment; B—Kruskal-Wallis and Dunn’s post hoc test revealing significant differences in the length of tegumental surface invaginations over the weeks following excystment.
(XLSX)

S1 Fig Survival and growth of juvenile *Fasciola hepatica* maintained *in vitro* in a variety of media. Fluke were maintained in RPMI, 5%, 10% or 20% Foetal Bovine Serum (FBS) in RPMI, NCTC, 5%, 10%, or 20% FBS in NCTC, *Fasciola* Saline, PBS and 100% FBS over 27 days following excystment. A—Percentage survival of juvenile *F*. *hepatica* over 27 weeks (mean±SEM) with statistical analyses performed using One Way ANOVA with Dunnett’s post hoc test and Kruskal-Wallis with Dunn’s post hoc test; B—Surface area of juvenile *F*. *hepatica* in mm^2^ (mean±SEM) with statistical analyses performed using Kruskal-Wallis with Dunn’s post hoc test and Mann Whitney-U test. *, P<0.05; ****, P<0.0001.
(TIF)

S2 Fig Significant fluke growth was not induced by supplementation of growth media with bovine serum albumin, fatty acids or palmitic acid. RPMI was supplemented with either a fatty acid mixture (FA), palmitic acid (PA) or bovine serum albumin (BSA) and growth compared to that displayed by worms maintained in unsupplemented RPMI and those maintained in RPMI+50% Chicken Serum (RPMI+CS). Each data-point represents a measurement from an individual worm. Red horizontal lines represent dataset mean, with statistical analysis assessed via One Way ANOVA with Dunnett’s post hoc test. Significance is indicated versus untreated sample (RPMI). ****, p<0.0001.
(TIFF)

S3 Fig Confocal microscope image of gonoduct/ventral sucker region in juvenile *Fasciola hepatica* grown for 29 weeks *in vitro*. The worm was grown in 20% CS in RPMI and displayed the development of reproductive system tubing (*) extending posteriorly from the gonoduct (G) underneath the ventral sucker (VS) towards the uterus (red indicates muscle actin staining following labelling with phalloidin-tetramethyl rhodamine isothiocyanate) (scale bar 50 μm).
(TIF)

S4 Fig Tegumental spine development in juvenile *Fasciola hepatica* grown *in vitro* for 29 weeks. Scanning electron microscope image showing the appearance of developed spines at the posterior dorsal surface of a juvenile fluke grown in 50% CS in RPMI (scale bar 5 μm).
(TIF)

S5 Fig Relative transcript abundance of ago and nanos orthologues in growing and non-growing *Fasciola hepatica*. Worms maintained for 4 days +/- chicken serum (growing or non-growing respectively) were measured by qPCR using cDNA pools normalised for RNA input. A—Log2 fold change in gene expression between growing and non-growing worms showed upregulation of ago-2 and nanos orthologues and of the positive control gene CaM2. The red, dashed line represents baseline/unchanged expression levels. B—Band intensity of ago (203 bp), nanos (302 bp), and CaM2 (106 bp) show upregulation in growing worms when amplicons are run on a 1% agarose gel. Note: low molecular weight bands present in nanos lanes represent primer dimers.
(TIFF)

S1 Video The typical probing movement displayed by migrating juvenile stage *Fasciola hepatica in vitro*. The fluke shown were maintained *in vitro* in RPMI with 50% Chicken Serum for 39 days.
(AVI)

S2 Video The typical irregular wave-like movement of non-migrating stage *Fasciola hepatica in vitro*. The fluke shown was maintained *in vitro* in RPMI with 50% Chicken Serum for 205 days.
(AVI)

## References

[1] Food and Agriculture Organisation of the United Nations. Diseases in Domestic Animals Caused by Flukes. Rome: Food and Agriculture Organisation; 1994.

[2] Gray GD, Copland RS, Copeman DB. Overcoming liver fluke as a constraint to ruminant production in South-East Asia. Aust Cent Int Agric Res. 2008.

[3] Mehra UR, Verma AK, Dass RS, Sharma RL, Yadav SC. Effects of *Fasciola gigantica* infection on growth and nutrient utilisation of buffalo calves. Vet Rec. 1999;145: 699–702. 10638797

[4] Tolan RW. Fascioliasis due to *Fasciola hepatica* and *Fasciola gigantica* infection: an update on this “Neglected” Neglected Tropical Disease. Lab Med. 2011;42: 107–116.

[5] Brennan GP, Fairweather I, Trudgett A, Hoey E, McCoy, McConville M, et al Understanding triclabendazole resistance. Exp Mol Pathol. 2007;82: 104–109. 17398281 10.1016/j.yexmp.2007.01.009

[6] Hodgkinson J, Cwiklinski K, Beesley NJ, Paterson S, Williams DJL. Identification of putative markers of triclabendazole resistance by a genome-wide analysis of genetically recombinant *Fasciola hepatica*. Parasitology. 2013;140: 1523–1533. 10.1017/S0031182013000528 23721579

[7] Fox NJ, White PCL, McClean CJ, Marion G, Evans A, Hutchings MR. Predicting impacts of climate change on *Fasciola hepatica* risk. PLoS One. 2011;6: e16126 10.1371/journal.pone.0016126 21249228 PMC3018428

[8] Cwiklinski K, Dalton JP, Dufresne PJ, La Course J, Williams DJ, Hodgkinson J, et al The *Fasciola hepatica* genome: gene duplication and polymorphism reveals adaptation to the host environment and the capacity for rapid evolution. Genome Biol. 2015;16: 71 10.1186/s13059-015-0632-2 25887684 PMC4404566

[9] Young ND, Hall RS, Jex AR, Cantacessi C, Gasser RB. Elucidating the transcriptome of *Fasciola hepatica*—a key to fundamental and biotechnological discoveries for a neglected parasite. Biotechnol Adv. Elsevier Inc.; 2010;28: 222–231. 10.1016/j.biotechadv.2009.12.003 20006979

[10] Young ND, Jex AR, Cantacessi C, Hall RS, Campbell BE, Spithill TW, et al A portrait of the transcriptome of the neglected trematode, *Fasciola gigantica—*biological and biotechnological implications. PLoS Negl Trop Dis. 2011;5: e1004 10.1371/journal.pntd.0001004 21408104 PMC3051338

[11] McGonigle L, Mousley A, Marks NJ, Brennan GP, Dalton JP, Spithill TW, et al The silencing of cysteine proteases in *Fasciola hepatica* newly excysted juveniles using RNA interference reduces gut penetration. Int J Parasitol. 2008;38: 149–155. 18048044 10.1016/j.ijpara.2007.10.007

[12] Rinaldi G, Morales ME, Cancela M, Castillo E, Brindley PJ, Tort JF. Development of functional genomic tools in trematodes: RNA interference and luciferase reporter gene activity in *Fasciola hepatica*. PLoS Negl Trop Dis. 2008;2: e260 10.1371/journal.pntd.0000260 18612418 PMC2440534

[13] McVeigh P, McCammick EM, McCusker P, Morphew RM, Mousley A, Abidi A, et al RNAi dynamics in juvenile *Fasciola* spp. liver flukes reveals the persistence of gene silencing *in vitro*. PLoS Negl Trop Dis. 2014;8: e3185 10.1371/journal.pntd.0003185 25254508 PMC4177864

[14] Dell’Oca N, Basika T, Corvo I, Castillo E, Brindley PJ, Rinaldi G, et al RNA interference in *Fasciola hepatica* newly excysted juveniles: Long dsRNA induces more persistent silencing than siRNA. Mol Biochem Parasitol. 2014;197: 28–35. 10.1016/j.molbiopara.2014.10.001 25307443

[15] Lacourse EJ, Perally S, Hernandez-Viadel M, Wright HA, Brophy PM. A proteomics approach to quantify protein levels following RNA interference: case study with glutathione transferase superfamily from the model metazoan *Caenorhabditis elegans*. J Proteome Res. 2008;7: 3314–3318. 10.1021/pr8001035 18582093

[16] Krautz-Peterson G, Radwanska M, Ndegwa D, Shoemaker CB, Skelly PJ. Optimizing gene suppression in schistosomes using RNA interference. Mol Biochem Parasitol. 2007;153: 194–202. 17420062 10.1016/j.molbiopara.2007.03.006

[17] He Y, Cai G, Ni Y, Li Y, Zong H, He L. siRNA-mediated knockdown of two tyrosinase genes from *Schistosoma japonicum* cultured *in vitro*. Exp Parasitol. 2012;132: 394–402. 10.1016/j.exppara.2012.10.001 23073288

[18] Sripa J, Pinlaor P, Brindley PJ, Sripa B, Kaewkes S, Robinson MW, et al RNA interference targeting cathepsin B of the carcinogenic liver fluke, *Opisthorchis viverrini*. Parasitol Int. 2011;60: 283–288. 10.1016/j.parint.2011.04.003 21565281 PMC3682765

[19] Wang X, Chen W, Tian Y, Huang Y, Li X, Yu X. RNAi-mediated silencing of enolase confirms its biological importance in *Clonorchis sinensis*. Parasitol Res. 2014;113: 1451–1458. 10.1007/s00436-014-3785-0 24458653

[20] Wikerhauser T, Cvetnić S. Survival of young and sexually mature adult *Fasciola hepatica* in various cell-free media with and without mammalian cell cultures. Exp Parasitol. 1967;20: 200–204. 4962448 10.1016/0014-4894(67)90039-2

[21] Wikerhauser T, Cvetnić S, Brudnjak Z. Further study of the survival of young *Fasciola hepatica* in cell cultures. Wiadomości Parazytol. 1968;14: 703–705. 5712599

[22] Osuna Carrillo de Albornoz A, y Guevara Pozo D. Cultivo de Helmintos Parasitos. I. Primeros resultados con un medio basico para el cultivo “*in vitro*” de *Fasciola hepatica*. Rev Iber Parasitol. 1974;34: 1–2.

[23] Davies C, Smyth JD. *In vitro* cultivation of *Fasciola hepatica* metacercariae and of partially developed flukes recovered from mice. Int J Parasitol. 1978;8: 125–131. 681068 10.1016/0020-7519(78)90006-1

[24] Smith MA, Clegg JA. Improved culture of *Fasciola hepatica in vitro*. Z Parasitenkd. 1981;66: 9–15. 7198848 10.1007/BF00941940

[25] Cancela M, Acosta D, Rinaldi G, Silva E, Durán R, Roche L, et al A distinctive repertoire of cathepsins is expressed by juvenile invasive *Fasciola hepatica*. Biochimie. 2008;90: 1461–1475. 10.1016/j.biochi.2008.04.020 18573308

[26] Beckham SA, Piedrafita D, Phillips CI, Samarawickrema N, Law RHP, Smooker PM, et al A major cathepsin B protease from the liver fluke *Fasciola hepatica* has atypical active site features and a potential role in the digestive tract of newly excysted juvenile parasites. Int J Biochem Cell Biol. 2009;41: 1601–1612. 10.1016/j.biocel.2009.02.003 19401154 PMC3514016

[27] Robinson MW, Menon R, Donnelly SM, Dalton JP, Ranganathan S. An integrated transcriptomics and proteomics analysis of the secretome of the helminth pathogen *Fasciola hepatica*: proteins associated with invasion and infection of the mammalian host. Mol Cell Proteomics. 2009;8: 1891–1907. 10.1074/mcp.M900045-MCP200 19443417 PMC2722771

[28] Collins JJ, Wendt GR, Iyer H, Newmark PA. Stem cell progeny contribute to the schistosome host-parasite interface. Elife. 2016;22: e12473. 10.7554/eLife.12473 PMC4841766 27003592

[29] Ndegwa D, Krautz-Peterson G, Skelly PJ. Protocols for gene silencing in schistosomes. Exp Parasitol. 2007;117: 284–91. 17870072 10.1016/j.exppara.2007.07.012 PMC2693101

[30] Dawes B. On the growth and maturation of *Fasciola hepatica* L. in the mouse. J Helminthol. 1962;36: 11–38. 13884021 10.1017/s0022149x00022343

[31] Bennett CEE, Threadgold LTT. *Fasciola hepatica*: development of tegument during migration in mouse. Exp Parasitol. 1975;38: 38–55. 1149868 10.1016/0014-4894(75)90036-3

[32] Wang B, Collins JJ, Newmark PA. Functional genomic characterization of neoblast-like stem cells in larval *Schistosoma mansoni*. Elife. 2013;2: e00768 10.7554/eLife.00768 23908765 PMC3728622

[33] Skog S, Tribukait B, Wallstrom B, Eriksson S. Hydroxyurea-induced cell death as related to cell cycle in mouse and human T-lymphoma cells. Cancer Res. 1987;47: 6490–6493. 3499975

[34] Rossi L, Salvetti A, Lena A, Batistoni R, Deri P, Pugliesi C, Loreti E, Gremigni V. DjPiwi-1, a member of the PAZ-Piwi gene family, defines a subpopulation of planarian stem cells. Dev Genes Evol. 2006;216: 335–346. 16532341 10.1007/s00427-006-0060-0

[35] Collins JJ, Wang B, Lambrus BG, Tharp ME, Iyer H, Newmark PA. Adult somatic stem cells in the human parasite *Schistosoma mansoni*. Nature. 2013;494: 476–9. 10.1038/nature11924 23426263 PMC3586782

[36] Ladurner P, Reiger R, Baguña J. Spatial distribution and differentiation potential of stem cells in hatchlings and adults in the marine platyhelminth *Macrostomum* sp.: A bromodeoxyuridine analysis. Dev Biol. 2000;226: 231–241. 11023683 10.1006/dbio.2000.9867

[37] Fredensborg BL, Poulin R. *In vitro* cultivation of *Maritrema novaezealandensis* (Microphallidae): the effect of culture medium on excystation, survival and egg production. Parasitol Res. 2005;95: 310–313. 15682336 10.1007/s00436-004-1293-3

[38] Lloyd MM, Poulin R. *In vitro* culture of marine trematodes from their snail first intermediate host. Exp Parasitol. 2011;129: 101–106. 10.1016/j.exppara.2011.07.009 21801722

[39] West J, Mitchell A, Pung OJ. Optimization of conditions for *in vitro* culture of the Microphallid Digenean *Gynaecotyla adunca*. J Parasitol Res. 2014: 382153 10.1155/2014/382153 24795817 PMC3984872

[40] Khaki Z, Khazraiinia P, Chegini S, Khazraee Nia S. Comparative study of serum lipid profile in chicken, ostrich, cattle, and sheep. Comp Clin Path. 2010;21: 259–263.

[41] Timanova-Atanasova A, Jordanova R, Radoslavov G, Deevska G, Bankov I, Barrett J. A native 13-kDa fatty acid binding protein from the liver fluke *Fasciola hepatica*. Biochim Biophys Acta. 2004;1674: 200–4. 15374624 10.1016/j.bbagen.2004.06.018

[42] Bennett CEE. Surface features, sensory structures, and movement of the newly excysted juvenile *Fasciola hepatica* L. J Parasitol. 1975;61: 886–891. 1185430

[43] Bennett CEE. Scanning electron microscopy of *Fasciola hepatica* L. during growth and maturation in the mouse. J Parasitol. 1975;61: 892–898. 1185431

[44] Baguñà J. The planarian neoblast: the rambling history of its origin and some current black boxes. Int J Dev Biol. 2012;56: 19–37. 10.1387/ijdb.113463jb 22252540

[45] Koziol U, Rauschendorfer T, Zanon Rodríguez L, Krohne G, Brehm K. The unique stem cell system of the immortal larva of the human parasite *Echinococcus multilocularis*. Evodevo. 2014;5: 10 10.1186/2041-9139-5-10 24602211 PMC4015340

[46] Morphew RM, Wright HA, LaCourse EJ, Woods DJ, Brophy PM. Comparative proteomics of excretory-secretory proteins released by the liver fluke *Fasciola hepatica* in sheep host bile and during *in vitro* culture ex host. Mol Cell Proteomics. 2007;6: 963–972. 17308300 10.1074/mcp.M600375-MCP200

[47] Robinson MW, Tort JF, Lowther J, Donnelly SM, Wong E, Xu W, et al Proteomics and phylogenetic analysis of the cathepsin L protease family of the helminth pathogen *Fasciola hepatica*: expansion of a repertoire of virulence-associated factors. Mol Cell Proteomics. 2008;7: 1111–1123. 10.1074/mcp.M700560-MCP200 18296439

