# Supplementary figures and images for: Stimulating Neoblast-Like Cell Proliferation in Juvenile Fasciola hepatica Supports Growth and Progression towards the Adult Phenotype In Vitro
Source: PLoS Negl Trop Dis. 2016 Sep 13;10(9):e0004994. doi: 10.1371/journal.pntd.0004994 (PMC5021332; doi:10.1371/journal.pntd.0004994)

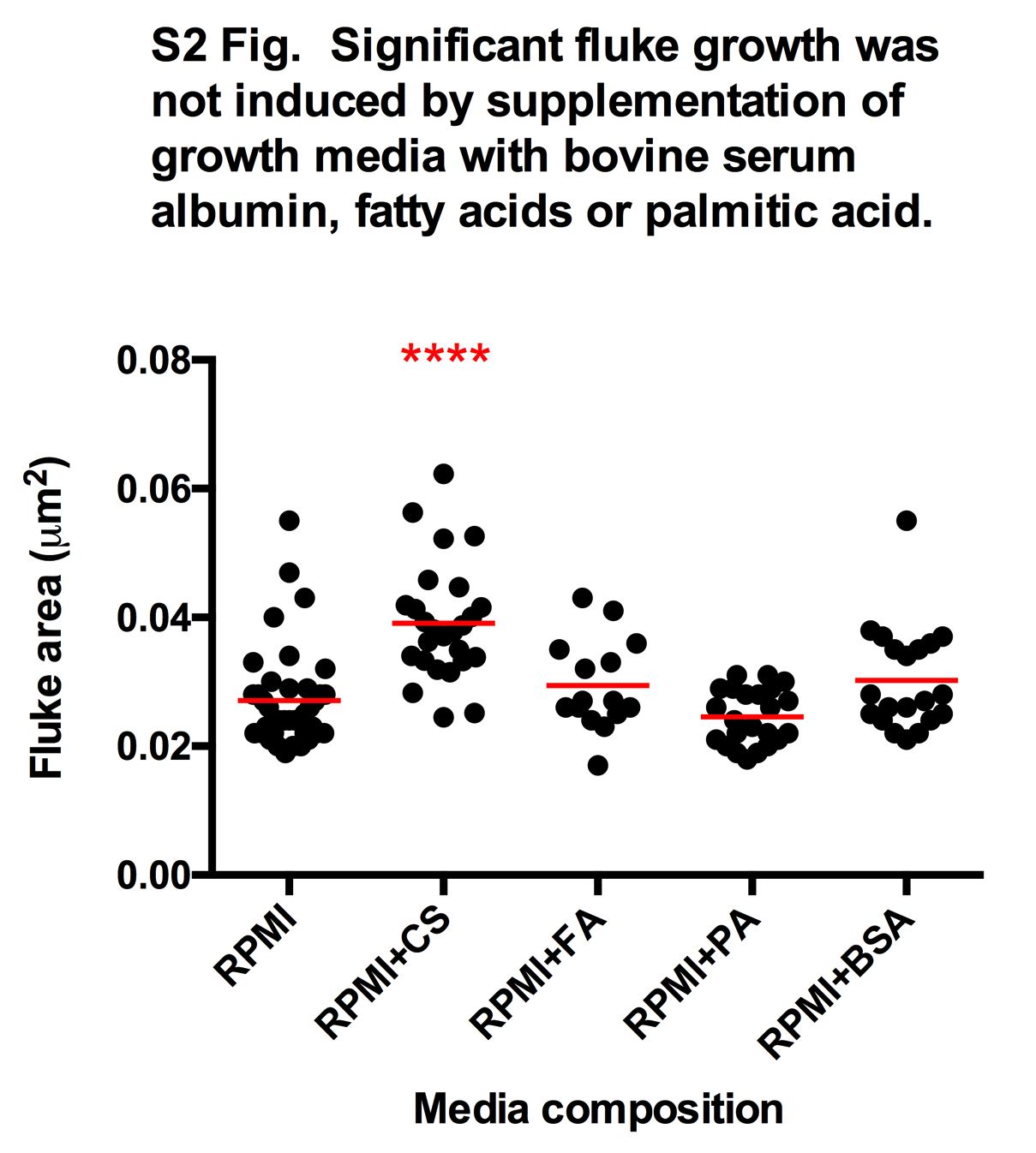

Supplement: S2 Fig — RPMI was supplemented with either a fatty acid mixture (FA), palmitic acid (PA) or bovine serum albumin (BSA) and growth compared to that displayed by worms maintained in unsupplemented RPMI and those maintained in RPMI+50% Chicken Serum (RPMI+CS). Each data-point represents a measurement from an individual worm. Red horizontal lines represent dataset mean, with statistical analysis assessed via One Way ANOVA with Dunnett’s post hoc test. Significance is indicated versus untreated sample (RPMI). ****, p<0.0001. (TIFF) [file pntd.0004994.s006.tiff]

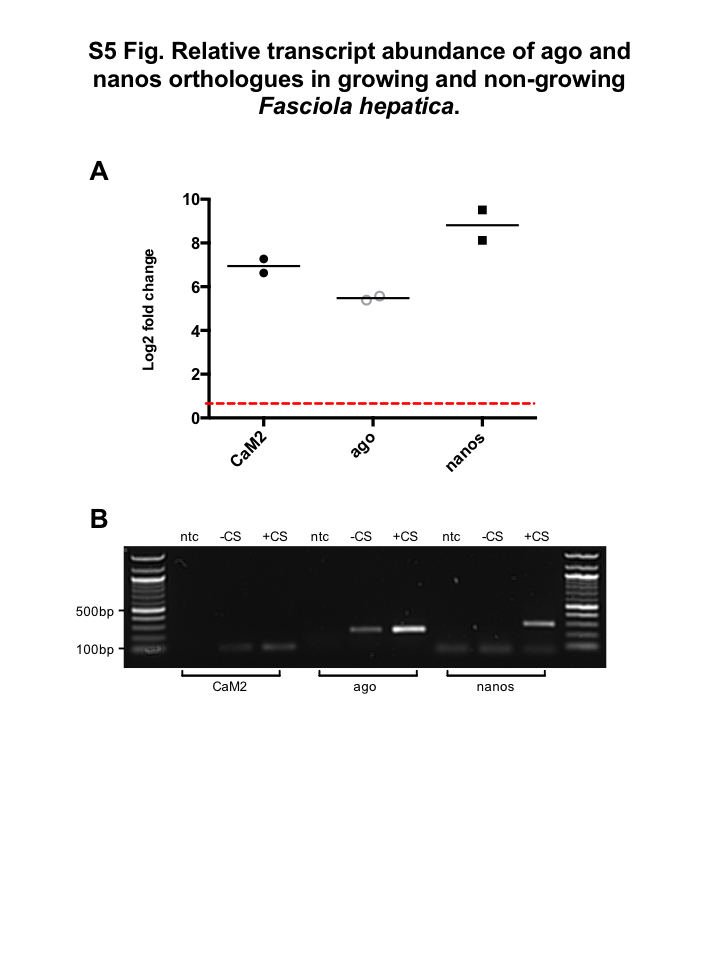

Supplement: S5 Fig — Worms maintained for 4 days +/- chicken serum (growing or non-growing respectively) were measured by qPCR using cDNA pools normalised for RNA input. A—Log2 fold change in gene expression between growing and non-growing worms showed upregulation of ago-2 and nanos orthologues and of the positive control gene CaM2. The red, dashed line represents baseline/unchanged expression levels. B—Band intensity of ago (203 bp), nanos (302 bp), and CaM2 (106 bp) show upregulation in growing worms when amplicons are run on a 1% agarose gel. Note: low molecular weight bands present in nanos lanes represent primer dimers. (TIFF) [file pntd.0004994.s009.tiff]
